# Pharmacokinetics and pharmacodynamics of VEGF-neutralizing antibodies

**DOI:** 10.1186/1752-0509-5-193

**Published:** 2011-11-21

**Authors:** Stacey D Finley, Marianne O Engel-Stefanini, PI Imoukhuede, Aleksander S Popel

**Affiliations:** 1 Department of Biomedical Engineering, Johns Hopkins University, School of Medicine, 720 Rutland Avenue, Baltimore, MD 21205, USA

## Abstract

**Background:**

Vascular endothelial growth factor (VEGF) is a potent regulator of angiogenesis, and its role in cancer biology has been widely studied. Many cancer therapies target angiogenesis, with a focus being on VEGF-mediated signaling such as antibodies to VEGF. However, it is difficult to predict the effects of VEGF-neutralizing agents. We have developed a whole-body model of VEGF kinetics and transport under pathological conditions (in the presence of breast tumor). The model includes two major VEGF isoforms VEGF_121 _and VEGF_165_, receptors VEGFR1, VEGFR2 and co-receptors Neuropilin-1 and Neuropilin-2. We have added receptors on parenchymal cells (muscle fibers and tumor cells), and incorporated experimental data for the cell surface density of receptors on the endothelial cells, myocytes, and tumor cells. The model is applied to investigate the action of VEGF-neutralizing agents (called "anti-VEGF") in the treatment of cancer.

**Results:**

Through a sensitivity study, we examine how model parameters influence the level of free VEGF in the tumor, a measure of the response to VEGF-neutralizing drugs. We investigate the effects of systemic properties such as microvascular permeability and lymphatic flow, and of drug characteristics such as the clearance rate and binding affinity. We predict that increasing microvascular permeability in the tumor above 10^-5 ^cm/s elicits the undesired effect of increasing tumor interstitial VEGF concentration beyond even the baseline level. We also examine the impact of the tumor microenvironment, including receptor expression and internalization, as well as VEGF secretion. We find that following anti-VEGF treatment, the concentration of free VEGF in the tumor can vary between 7 and 233 pM, with a dependence on both the density of VEGF receptors and co-receptors and the rate of neuropilin internalization on tumor cells. Finally, we predict that free VEGF in the tumor is reduced following anti-VEGF treatment when VEGF_121 _comprises at least 25% of the VEGF secreted by tumor cells.

**Conclusions:**

This study explores the optimal drug characteristics required for an anti-VEGF agent to have a therapeutic effect and the tumor-specific properties that influence the response to therapy. Our model provides a framework for investigating the use of VEGF-neutralizing drugs for personalized medicine treatment strategies.

## Background

Angiogenesis, the formation of new capillaries from pre-existing blood vessels, is a tightly regulated biological process and is involved in normal physiological function as well as in pathological conditions. Angiogenesis occurs in embryos during organ growth and development [1]. In adults, angiogenesis is essential for conditions requiring an increase in blood and oxygen supply, including reproduction, physiological repair (e.g., wound and tissue healing), and exercise [2,3]. In addition to its relevance in physiological conditions, angiogenesis has a prominent role in diseases such as preeclampsia, ischemic heart disease, and cancer. Neovascularization allows for cancer development, tumor growth, and metastasis whereby the tumor elicits the formation of capillaries to obtain its own blood supply [4].

Vascular endothelial growth factor (VEGF) is a potent regulator of angiogenesis, and its role in cancer biology has been widely studied. Clinically, cancer patients exhibit increased VEGF levels [5]¸ although this finding remains controversial [6], and vascularization in tumors shows marked differences from physiological vessel architecture: increased leakiness and tortuosity, decreased pericyte coverage, and abnormal organization [7,8]. For these reasons, many cancer therapies target angiogenic pathways, with the major focus being on VEGF-mediated signaling in the form of antibodies to VEGF and its receptors, small molecule tyrosine kinase inhibitors, and peptides [9-11].

The human VEGF family includes five ligands (VEGF-A through -D and placental growth factor, PlGF), three receptors (VEGFR1, VEGFR2, and VEGFR3), and two co-receptors, neuropilins (NRP1 and NRP2). VEGF binding to its receptors regulates vessel permeability [12] and expression of matrix metalloproteinases [13], involved in capillary sprout formation. Angiogenesis involves numerous molecular species and includes events that occur at the molecular, cellular, and tissue levels in sequence and in parallel. This complexity lends the process of angiogenesis to systems biology approaches [14,15]. Computational modeling, in particular, is useful in understanding angiogenesis and provides a framework to test biological hypotheses [16]. Additionally, the models can aid in the development and optimization of therapies targeting this process [16-19].

Our laboratory previously developed a whole-body model of VEGF kinetic and transport necessary for building models of VEGF-mediated angiogenesis [20,21]. One of the models predicts the distribution of VEGF in the body upon administration of the anti-VEGF recombinant humanized monoclonal antibody bevacizumab [21]. The findings suggest that anti-VEGF agents act to deplete tumor VEGF rather than blood (plasma) VEGF because the blood VEGF was predicted to decrease transiently and then increase above the baseline pre-treatment level. In the present study, we extend the previous computational model to include receptors on parenchymal cells. Our previous models were limited by a lack of quantitative measurements of cell surface receptor densities. Therefore, using quantitative flow cytometry, we have determined the density of VEGF receptors and co-receptors on the surface of endothelial cells, skeletal muscle myocytes, and tumor cells, and incorporated these key parameters into the current model. Additionally, we have included VEGF degradation and have utilized published *in vitro *data to establish a baseline for the rate of VEGF secretion by tumor cells. These significant model additions provide a physiologically-based computational framework to study VEGF kinetics and transport.

We utilize the model to investigate how systemic properties, drug characteristics, and properties of the tumor microenvironment influence the response to the anti-VEGF agent. The simulations show that the level of VEGF in the tumor interstitium can decrease or, paradoxically, increase beyond even the baseline pre-treatment level as a result of anti-VEGF administration depending on the values of parameters. Importantly, we predict the ranges of parameter values which elicit the undesired effect of increasing tumor interstitial VEGF concentration. Thus, our model can be used to predict the optimal drug and tumor properties for which an anti-VEGF agent may have a therapeutic effect.

## Methods

### Computational methods

#### Computational Model

Here, we summarize the features of the model and describe significant enhancements from previous models: the presence of VEGF receptors on parenchymal cells, incorporating experimental quantification of VEGF receptor expression, and the degradation of VEGF in the tissue compartments; the degradation of VEGF in the blood compartment is included in the clearance term. The complete set of 67 ordinary differential equations (ODEs) is given in Additional file 1, and prior models created by our laboratory are fully detailed in our previous papers [20,21].

The model is comprised of three compartments: normal tissue ("normal compartment", represented by skeletal muscle), the vascular system ("blood compartment"), and diseased tissue ("tumor compartment"). As a starting point, we modeled a breast tumor measuring 4 cm in diameter; however, the tumor compartment can be adapted to represent any solid tumor in the body, including metastatic disease. Thus, the setting mimics neoadjuvant therapy when the agent is administered prior to surgical rescission of the tumor; alternatively, the tumor may represent a metastatic growth.

The tissue is divided into parenchymal cells, capillaries, and interstitial space. Parenchymal cells include muscle fibers in the normal compartment and tumor cells in the tumor compartment. The interstitial space is subdivided into the extracellular matrix (ECM), the endothelial cell basement membrane (EBM), and parenchymal cell basement membrane (PBM). The two VEGF isoforms included in the model, VEGF_121 _and VEGF_165_, are secreted from the parenchymal cells and can diffuse in the interstitium. VEGF_165 _contains a heparin-binding domain that enables this isoform to bind reversibly to glycosaminoglycan (GAG) chains in the ECM and basement membranes; in contrast, VEGF_121 _can diffuse freely in the interstitial fluid. A portion of the interstitial space is unavailable to VEGF because of the volume occupied by the extracellular matrix molecules and closed pores in the matrix. Therefore, we specify *U_AV_*, the available fluid volume, as *U_AV _*= *K_AV _*× *U*, where *K_AV _*is the available volume fraction, and *U *is the tissue volume.

**Figure 1:**
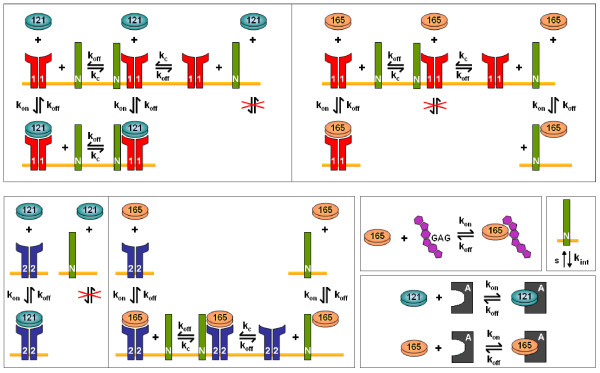
**Molecular interactions**. The binding interactions between VEGF, surface receptors, extracellular matrix and basement membranes. VEGF_165 _binds to VEGFR1, VEGFR2, and co-receptors NRP1 and NRP2. VEGF_165 _also binds to glycosaminoglycan (GAG) chains in the extracellular matrix and basement membranes. VEGF_121 _binds to VEGFR1 and VEGFR2, but is unable to bind to NRPs. The molecular interactions between the VEGF isoforms and NRP1 or NRP2 are identical, but are governed by different kinetic parameters. The anti-VEGF agent binds to both isoforms. The receptors and co-receptors are inserted and internalized at the cell surface.

The molecular interactions of VEGF and its receptors are illustrated in Figure 1. This model includes the presence of VEGF receptors VEGFR1 and VEGFR2, as well as co-receptors neuropilins NRP1 and NRP2. VEGF binds to receptors present on the luminal and abluminal endothelial surfaces, on muscle fibers, and on tumor cells. VEGFR1, VEGFR2, and NRP1 are all present on endothelial cells. Muscle fibers may express very low levels of VEGFR1 and VEGFR2; however, the density of NRP1 is relatively high. Additionally, tumor cells express both NRP1 and NRP2 [22] as well as VEGFR1 and VEGFR2. Ligated and unligated receptors are internalized; however, the total number of receptors of each type is assumed to be constant at any given time, i.e., as receptors are internalized, an equal number of unligated receptors is instantaneously inserted into the cell membrane. This assumption can be easily relaxed when information about receptor dynamics becomes available. Intercompartment transport of free VEGF includes vascular permeability and lymph flow. VEGF intravasates and extravasates via transendothelial macromolecular permeability. Additionally, VEGF can flow from the normal tissue into the blood via lymphatic drainage. However, it is assumed that tumor lymphatics are non-functioning, based on experimental evidence [23,24]. Lastly, the ligand can be removed from the blood via clearance.

In addition to disappearing via receptor-mediated internalization and blood clearance, VEGF is subject to proteolysis by plasmin and matrix metalloproteinases. Therefore, we have included an intrinsic protein degradation term for VEGF, which has not appeared in our previous models. Based on experimental measurements for the half-life of VEGF [25-27], we have set the degradation rate constant to be 1.93 × 10^-4 ^s^-1^, corresponding to a half-life of 60 minutes.

The model includes an anti-VEGF agent that is added to the blood compartment once steady-state is reached, simulating an intravenous injection. The anti-VEGF binds to both VEGF isoforms in all compartments to form the VEGF/anti-VEGF complex. Free anti-VEGF and the complex can be transported via microvascular permeability and lymphatic drainage, and can be cleared from the blood. Administration of the anti-VEGF occurs via infusion for 90 minutes at a dose of 10 mg/kg. In this study, we simulate a 70-kg patient; therefore, the rate at which the anti-VEGF is administered is 7.78 mg/min (denoted as *q_A _*in equation S.38 of Additional file 1).

#### Numerical implementation

The model is described by 67 non-linear ordinary differential equations: 24 for the normal compartments, 16 for the blood, and 27 for the tumor compartment. These equations were implemented in MATLAB (v7.11.0.584 R2010b, Mathworks) using the SimBiology toolbox. The steady-state and dynamic solutions were calculated using the Sundials solver. An absolute tolerance of 10^-9 ^was used, and the relative tolerance was set at 10^-20^.

#### Model parameters

The geometric parameters of the compartments, the kinetic constants governing the molecular interactions, and the initial concentrations are the same as those used in our previous model [21], with the exception of the receptor densities and degradation. In the present model, we include *in vivo *and *in vitro *quantification of VEGF receptors (experimental methods based on quantitative flow cytometry are described below), and the receptor densities, presented as dimerized receptors, are given in Table 1. We perform a sensitivity study to determine the effect of varying the receptor density, thus these values provide a starting point for our analysis. We are particularly interested in the effect of neuropilin density, as only *in vitro *data are available for the expression of NRP1, and the level of NRP2 expression has not been quantified.

**Table 1:** Receptor densities

	Measured parameters		Model parameters	
	Value	Unit	Value	Unit
**VEGFR1**				
Luminal EC (normal)	550	rec/EC	1.21 × 10^-1^	pmol/cm^3 ^tissue
Abluminal EC (normal)	550	rec/EC	9.86 × 10^-3^	pmol/cm^3 ^tissue
Luminal EC (diseased)	3,750	rec/EC	4.38 × 10^-4^	pmol/cm^3 ^tissue
Abluminal EC (diseased)	3,750	rec/EC	6.54 × 10^-2^	pmol/cm^3 ^tissue
Myocytes	0	rec/myocyte	0	pmol/cm^3 ^tissue
Tumor cells	1,100	rec/tumor cell	2.81 × 10^-1^	pmol/cm^3 ^tissue

**VEGFR2**				
Luminal EC (normal)	350	rec/EC	7.70 × 10^-2^	pmol/cm^3 ^tissue
Abluminal EC (normal)	350	rec/EC	6.28 × 10^-3^	pmol/cm^3 ^tissue
Luminal EC (diseased)	300	rec/EC	3.51 × 10^-5^	pmol/cm^3 ^tissue
Abluminal EC (diseased)	300	rec/EC	5.23 × 10^-3^	pmol/cm^3 ^tissue
Myocytes	0	rec/myocyte	0	pmol/cm^3 ^tissue
Tumor cells	550	rec/tumor cell	1.41 × 10^-1^	pmol/cm^3 ^tissue

**NRP1**				
Luminal EC (normal)	17,500	rec/EC	3.74	pmol/cm^3 ^tissue
Abluminal EC (normal)	17,500	rec/EC	3.05 × 10^-1^	pmol/cm^3 ^tissue
Luminal EC (diseased)	20,000^†^	rec/EC	2.34 × 10^-3^	pmol/cm^3 ^tissue
Abluminal EC (diseased)	20,000^†^	rec/EC	3.49 × 10^-1^	pmol/cm^3 ^tissue
Myocytes	34,500	rec/myocyte	2.06	pmol/cm^3 ^tissue
Tumor cells	39,500	rec/tumor cell	1.01 × 10^1^	pmol/cm^3 ^tissue

**NRP2**				
Luminal EC (normal)	0	rec/EC	0	pmol/cm^3 ^tissue
Abluminal EC (normal)	0	rec/EC	0	pmol/cm^3 ^tissue
Luminal EC (diseased)	0	rec/EC	0	pmol/cm^3 ^tissue
Abluminal EC (diseased)	0	rec/EC	0	pmol/cm^3 ^tissue
Myocytes	0	rec/myocyte	0	pmol/cm^3 ^tissue
Tumor cells	39,500^‡^	rec/tumor cell	1.02 × 10^1^	pmol/cm^3 ^tissue

EC = endothelial cell; rec = receptors

^† ^Extrapolated from receptor density on normal ECs, accounting for different cell surface areas

^‡^No NRP2 quantification available; assumed to be the same as NRP1 on tumor cells

Conversions of receptor densities to tissue concentrations:

Abluminal EC receptors (normal): 1.79 × 10^-5 ^(pmol/cm^3 ^tissue)/(rec/EC);

Luminal EC receptors (normal): 2.20 × 10^-4 ^(pmol/cm^3 ^tissue)/(rec/EC);

Abluminal EC receptors (diseased): 1.74 × 10^-5 ^(pmol/cm^3 ^tissue)/(rec/EC);

Luminal EC receptors (diseased): 1.17 × 10^-7 ^(pmol/cm^3 ^tissue)/(rec/EC);

Myocyte receptors: 5.96 × 10^-5 ^(pmol/cm^3 ^tissue)/(rec/myocyte);

Tumor receptors: 2.55 × 10^-4 ^(pmol/cm^3 ^tissue)/(rec/tumor cell);

Myocyte receptor densities are expressed per myonuclear domain

### Experimental methods

#### Cell culture

Human umbilical vein endothelial cells (HUVEC), human dermal microvascular endothelial cells (MEC), and human skeletal muscle cells from gastrocnemius (SkM) were acquired from individual donors (Lonza, Walkersville, MD and Stem Cell Technologies, Vancouver, Canada). The endothelial cells were maintained in Endothelial Cell Growth Medium-2 (EGM-2), supplemented by the EGM-2 SingleQuot Kit for HUVECs, or supplemented by the EGM-2 Microvascular SingleQuot Kit for MECs (Lonza). The skeletal muscle cells were maintained in Skeletal Muscle Cell Growth Medium (SkGM) supplemented by the SkGM BulletKit (Lonza). Breast cancer cells MDA-MB-231were kindly provided by Dr. Zaver M. Bhujwalla (Johns Hopkins University) with the following details about the cell line: MDA-MB-231 breast cancer cells were purchased from the American Type Culture Collection (ATCC) and used within 6 months of obtaining them from ATCC; the cell line was tested and authenticated by ATCC by two independent methods; the ATCC cytochrome C oxidase I PCR assay and short tandem repeat profiling using multiplex PCR. MDA-MB-231 cells were maintained in DMEM containing 10% fetal bovine serum (Invitrogen) and 1% Penicillin-Streptomycin (Invitrogen). Cells were grown at 37°C in 95% air, 5% CO_2_. Cells were grown to confluence before use and primary cells were only used through passage 6. For routine cell culture, cells were detached from flasks using 0.25% TrypLE (Invitrogen, Carlsbad, CA).

#### Tumor xenograft

Animal protocols were approved by the Institutional Care and Use Committee at the Johns Hopkins Medical Institutions (JHMI). MDA-MB-231 cells were dissociated from flasks with TrypLE (Invitrogen, Carlsbad, CA), washed twice in PBS, and resuspended in Dulbecco's Modified Eagle Medium. Mice were anesthetized using 0.125 mg Acepromazine and 12.5 mg Ketamine. Subsequently, 2 million cells/100-μL solution were injected into each side of the mammary fat pad of 7 week-old, female, athymic NCr-*nu/nu *mice. Tumors were grown for 23 days, to an average size of 620 ± 170 mm^3^, as calculated by measuring the long (*l*) and short (*s*) axis of the ellipsoid tumor with a caliper and applying the following equation: *V *= s^2^*l/2.

#### Endothelial cell isolation from tissue

Tissue was digested as previously described [28-30]. Briefly, tissue was minced into 1 mm sections and added to freshly prepared 0.2% collagenase type IV filtered (Worthington Biochemical Corporation, Lakewood, NJ), which had been reconstituted in Hanks Balanced Salt Solution without calcium and without magnesium. The tissue was digested for 30 min at 37°C with intermittent vortexing then passed through a 70 μm strainer (BD). Cells were centrifuged at 300 × g for 5 minutes and re-suspended in 30 mL of 0.2 μm filtered Isolation Buffer containing PBS without calcium and magnesium (Invitrogen), 2 mM EDTA (Mediatech), and 0.1% BSA (Sigma). Endothelial cells were isolated from the cell suspension using DSB-X (Invitrogen) biotinylated mouse CD31 antibody (eBioscience and BD Bioscience, San Diego, CA) and FlowComp Dynabeads (Invitrogen) according to the manufacturers' instructions.

In this study we only quantify VEGFR1 and VEGFR2, because the levels of these receptors are unchanged by the collagenase IV tissue dissociation; however, NRP1 is not quantified, because its surface levels are significantly decreased following collagenase IV treatment, due to the presence of trypsin [31]. Cell staining and flow cytometry were performed as we have previously described [31].

## Results

### Tumor interstitial free VEGF is sensitive to kinetic parameters for NRP2 molecular interactions

The current model includes NRP1 on muscle cells, and both NRP1 and NRP2 on tumor cells, which have not appeared in previous models. Given the range of values for the binding constant for NRP2 and VEGF_165 _and the lack of data for the coupling rate of NRP2 and VEGFR1 or VEGFR2, we performed a sensitivity study to determine the effect of these parameters (Additional file 2). In the sensitivity study, we vary each of these parameters individually over eight orders of magnitude and use the model to predict the steady-state free VEGF levels in the normal, blood, and tumor compartments. We calculate the percent change in free VEGF for the range of parameter values examined to determine how sensitive the model predictions are to the parameters of interest. We used the values of the kinetic parameters for NRP1 interactions as the starting baseline value (circles). Tumor VEGF is sensitive to the association rate for NRP2 and VEGF_165_, *k*_*on*,*V*165,*N*2 _(varies 22% for the range of parameter values examined), and we set this value to be 10^6 ^M^-1 ^s^-1^, which is in the range of available experimental data [32,33] (squares and triangles). We assumed the dissociation rate to be the same as VEGF_165 _dissociating from NRP1 (10^-3 ^s^-1^), resulting in a *K_d _*of 1 nM. We used the kinetic constants for NRP1 for *k*_*c*,*V*165*R*2,*N*2 _and *k*_*c*,*R*1,*N*2_, as the steady state concentrations are not sensitive to these parameters (less than 1% change in the free VEGF in all compartments). Although tumor free VEGF varies 18% across the range of values examined for *k*_*c*,*V*165*N*2,*R*2_, there is little experimental data available for this kinetic constant. Therefore, we set the value for the parameter to be the same as that for NRP1. All of the kinetic parameters governing the interactions of NRP2 are listed in Table 2.

**Table 2:** Kinetic constants for NRP2 interactions

	Measured parameters		Model parameters	
	Value	Unit	Value	Unit
***k***_*on,V*165,*N *2_	10^6^	M^-1 ^s^-1^	1.92 × 10^-3^	(pmol/cm^3 ^tissue)^-1 ^s^-1^
***k***_*c,V*165 *R*2,*N *2_	3.1 × 10^13^	(mol/cm^2^)^-1 ^s^-1^	2.02 × 10^-2^	(pmol/cm^3 ^tissue)^-1 ^s^-1^
***k***_*c,V*165 *N *2,*R*2_	10^14^	(mol/cm^2^)^-1 ^s^-1^	7.06 × 10^-2^	(pmol/cm^3 ^tissue)^-1 ^s^-1^
***k***_*c,R*1,*N *2_	10^14^	(mol/cm^2^)^-1 ^s^-1^	7.06 × 10^-2^	(pmol/cm^3 ^tissue)^-1 ^s^-1^

### The distribution and density of VEGF receptors on endothelial and parenchymal cells significantly alters free VEGF distributions in the normal tissue and tumor

**Figure 2:**
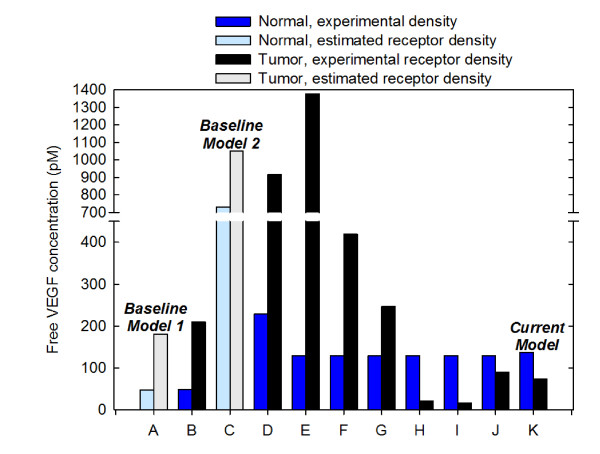
**Effect of model parameters**. The concentration of predicted free VEGF in the interstitium is sensitive to the presence and density of receptors on abluminal and luminal endothelial surfaces, and on myocytes and tumor cells. Baseline Model 1 is based on [20], and Baseline Model 2 is based on [34]. In the baseline models, receptor density is assumed to be: 10,000 VEGFR1, 10,000 VEGFR2, and 100,000 NRP1 molecules/endothelial cell. Experimental receptor density is based on *in vitro *in human cells using quantitative flow cytometry [31] and *in vivo *quantification in mouse skeletal muscle and tumor xenografts using the same technique. In each simulation, VEGF secretion is tuned to maintain blood free VEGF at 4.5 pM. Simulation cases are as follows: **A**, VEGFR1, VEGFR2, and NRP1 present on abluminal endothelial surface with assumed receptor density. **B**, VEGFR1, VEGFR2, and NRP1 present on abluminal endothelial surface with experimental receptor density. **C**, VEGFR1, VEGFR2, and NRP1 evenly distributed on abluminal and luminal endothelial surface with assumed receptor density. **D**, VEGFR1, VEGFR2, and NRP1 evenly distributed on abluminal and luminal endothelial surface with experimental receptor density. Cases E through K build upon the previous case by sequentially refining the model: **E**, Addition of NRP1 on myocytes. **F**, Addition of VEGFR1 on tumor cells. **G**, Addition of VEGFR2 present on tumor cells. **H**, Addition of NRP1 on tumor cells. **I**, Addition of NRP2 on tumor cells. **J**, Incorporation of VEGF degradation in normal tissue and tumor. **K**, Incorporation of experimental data for VEGF secretion by tumor cells (Current Model).

We have quantified the effect of utilizing experimental data for the density of VEGF receptors, accounting for VEGF receptors on both the abluminal and luminal endothelial surface, and including receptor expression on parenchymal cells (NRP1 on muscle fibers and VEGFR1, VEGFR2, NRP1, and NRP2 on tumor cells), as shown in Figure 2. In all cases, the plasma free VEGF concentration is fixed at 4.5 pM by readjusting the VEGF secretion rate. We have not included VEGF degradation in these simulations, in order to provide a fair comparison to previous models. We first compared our updated model to the model developed by Stefanini *et al. *[20] (referred to as Baseline Model 1), where VEGF receptors are expressed solely on the abluminal endothelial surface, and receptor density is assumed to be 10,000 VEGFR1, 10,000 VEGFR2, and 100,000 NRP1 molecules/endothelial cell. Experimental measurements of VEGF receptor expression are described in the Methods section, and the values are listed in Table 1. Incorporation of experimental data for the density of VEGF receptors results in an increase of 1% and 15% for free VEGF in the normal tissue and tumor, respectively, as compared to Baseline Model 1 (Figure 2, cases A and B). Thus, experimental quantification of VEGF receptors has a slight effect on the distribution of VEGF in the body, when considering only abluminal localization of receptors.

We next examined how the inclusion of luminal receptors impacts free VEGF in the body. Here, we compare to Baseline Model 2, based on a model by Stefanini and coworkers [34]. In Baseline Model 2, the receptor density is still assumed to be VEGFR1 = 10,000, VEGFR2 = 10,000, and NRP1 = 100,000 receptors/endothelial cell; however, receptors are now evenly distributed on the abluminal and luminal endothelial surface (i.e., for VEGFR1, there are 5,000 receptors on the luminal surface of the endothelial cells and 5,000 abluminal receptors). The equal distribution of receptors on the endothelial cell surface significantly increases free VEGF in the normal tissue and tumor (Baseline Model 1 compared to Baseline Model 2, i.e., Figure 2, case A compared to Figure 2, case C); free VEGF in the normal tissue increases more than 15-fold, while tumor VEGF increases almost 6-fold. This increase can be attributed to having to increase VEGF secretion in normal tissue and tumor in order to maintain 4.5 pM free VEGF in the plasma. Upon utilizing experimental data for VEGF receptor density and accounting for even distribution of receptors on the abluminal and luminal endothelial surfaces (Figure 2, case D), we found that the free VEGF in both the normal tissue and tumor decreased 3.2- and 1.1-fold, respectively, as compared to Baseline Model 2.

Lastly, we investigated the effect of parenchymal receptors on the concentration of free VEGF. We sequentially added VEGF receptors to different cell types and predicted the concentration of free VEGF in the normal tissue and tumor (Figure 2, cases E through I). In each case, VEGF secretion was tuned such that the concentration of free VEGF in the plasma was 4.5 pM. When NRP1 co-receptors are present on myocytes in the normal compartment, a significant change in the concentration of free VEGF in the normal tissue and tumor is observed (Figure 2, case E compared to Baseline Model 2). Free VEGF in the normal tissue is reduced almost 6-fold, since VEGF bound to the receptors on myocytes can be internalized, acting as a sink for VEGF. Conversely, tumor VEGF increases 1.3-fold. This is because extrapolation from *in vitro *experiments estimates the number of NRP1 co-receptors on the tumor endothelium to be 39,500 molecules/cell, compared to the value of 100,000 molecules/cell used in Baseline Model 2, leading to more unbound VEGF. As the density of VEGF receptors on tumor cells is adjusted to fully reflect experimental data by sequentially adding receptors, free VEGF in the tumor decreases 63-fold (Figure 2, case I) compared to Baseline Model 2 (Figure 2, case C). This analysis demonstrates the importance of having accurate estimates of receptor density, as the model is sensitive to these parameters.

### The rate of VEGF secretion and the VEGF isoform secretion ratio VEGF_165_:VEGF_121 _in the tumor affect the distribution of free VEGF in the body

**Figure 3:**
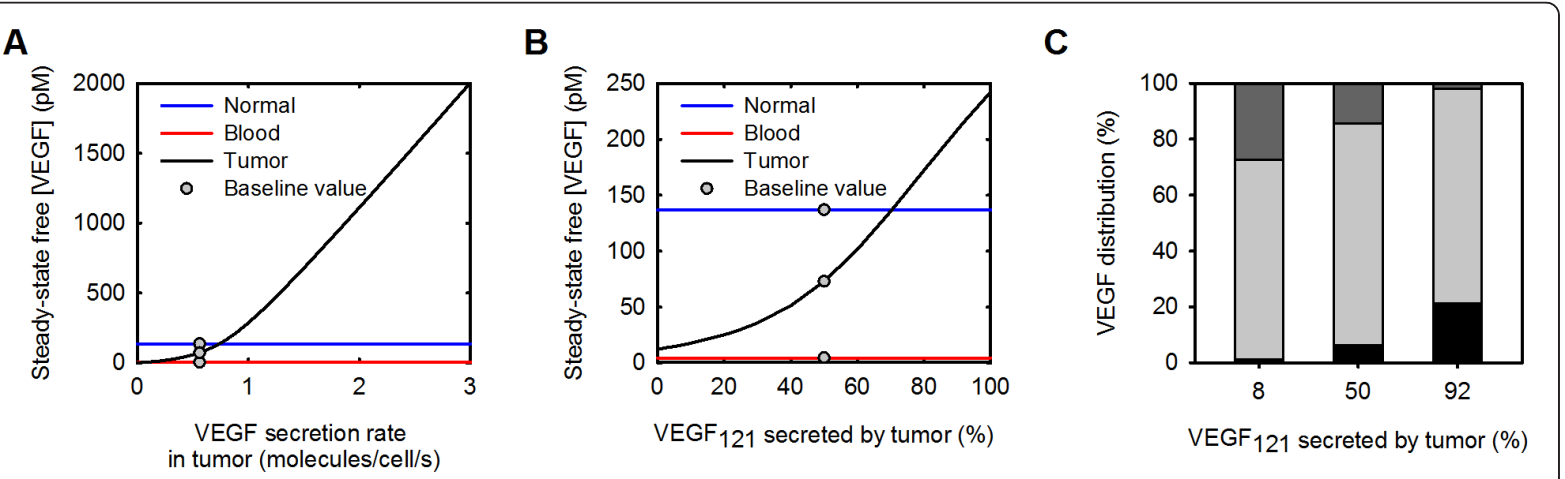
**Effect of tumor VEGF secretion**. The rate of VEGF secreted by tumor cells and the tumor isoform secretion ratio VEGF_165_:VEGF_121 _induces whole-body changes in the distribution of VEGF. **A**, Effect of tumor VEGF secretion rate on the steady-state free VEGF concentration in the body. Gray circles indicate the secretion rate used in the current model, 0.56 molecules/cell/s. **B**, Steady-state free VEGF concentration in the body. Gray circles indicate the ratio used in the current model, VEGF_165_:VEGF_121 _= 50%:50% in the tumor. **C**, Distribution of free, receptor-, and matrix-bound VEGF: black, unbound VEGF; light gray, receptor-bound VEGF; dark gray, VEGF bound to GAG chains in the extracellular matrix and basement membranes.

An important model parameter is the rate at which VEGF is secreted by muscle fibers in the normal tissue and tumor cells in the tumor compartment, which influences the level of free VEGF in the body. In previous models, we assumed that the parenchymal cells in the normal tissue and tumor secrete the same amount of VEGF. However, in the present study, we have incorporated *in vitro *measurements of the rate of VEGF secretion from cells from various human tumor cell lines [35-38]. Given the wide range of values for VEGF secretion (0.04 to 2.65 molecules/cell/s), we estimated the effect of the tumor VEGF secretion rate on steady-state free VEGF in the body (Figure 3A). When the rate of VEGF secreted by tumor cells is varied from 0 up to 3 molecules/cell/s, free VEGF in the tumor increases from 0.06 pM to 2 nM, an increase of more than four orders of magnitude. Conversely, free VEGF in the normal tissue and blood increase by 0.1% and 9%, respectively. These results show that varying the rate of VEGF secreted by tumor cells is a means of tuning the level of free VEGF in the tumor, leaving free VEGF in the normal tissue and blood largely unaffected. In this way, we are able to set the tumor secretion rate in order to achieve a certain level of free VEGF in the tumor. A detailed comparison to experimental data for tumor VEGF concentration is presented in Additional file 3. Briefly, we utilize a meta-analysis of VEGF content in cancer patients for various tumor types performed by Kut *et al. *[5]; the weighted average of intracellular and extracellular tumor VEGF for all cancer studies was reported to be 334 pg/mg protein, with a significant variation between different studies. This value represents the sum of the free and bound VEGF. Converting to the concentration of free VEGF requires the fraction of free:total VEGF, which depends of the isoform secretion ratio as well as the density of ECM binding sites and the number of VEGF cell surface receptors. For example, at 2% of free VEGF as a percent of total (free and bound), the above experimental value is equivalent to 82 pM for the free interstitial VEGF concentration; at 6% the concentration of free VEGF is 245 pM. Thus, the experimental data suggest that the interstitial tumor VEGF concentration levels are 10-60 times higher than the plasma concentration. Methods of isolating tumor interstitial fluid (TIF) exist that should allow measurements of protein concentration [39]; however, there is a lack of direct quantitative measurements of free VEGF in the TIF or in normal tissues such as skeletal muscle. As a baseline value, we have set the rate of VEGF secreted by tumor cells to be 0.56 molecules/cell/s (the average of the available experimental data), and this value is used in all simulations presented below. When VEGF is secreted at this rate, and VEGF degradation in the normal tissue and tumor compartments is included, free VEGF in the tumor is predicted to be 73 pM (Figure 2, case K).

In addition to the absolute rate at which VEGF is secreted by tumor cells, it is also important to consider the relative secretion rate of the VEGF isoforms. A compilation of qualitative information regarding the distribution of VEGF in tumor tissue reveals that VEGF_121 _is expressed at similar mRNA levels as, and in some cases at higher levels than, VEGF_165 _for several tumor types [40-44]. This is in contrast to skeletal muscle where VEGF_165 _expression is predominant [45,46]. Although these data are for mRNA expression, they represent the only quantitative data available for the relative levels of VEGF isoforms in tissue.

In order to understand the impact of the expression of VEGF isoforms, the isoform secretion ratio VEGF_165_:VEGF_121 _in the tumor was varied between 100%:0% to 0%:100% from the previous value of 92%:8%, while keeping the total VEGF secretion rate constant at 0.56 molecules/cell/s. The isoform secretion ratio in normal tissue remained at 92%:8%. As the relative amount of VEGF_121 _secreted by the tumor is increased from zero to 100%, free VEGF in the tumor increases by 94% (Figure 3B). This can be explained by examining the distribution of VEGF. Since VEGF_121 _is unable to bind to GAG chains in the extracellular matrix and basement membranes, the percentage of matrix-bound VEGF decreases as more VEGF_121 _is secreted (Figure 3C). Additionally, the VEGF internalization influences the free VEGF level. VEGF_165 _can bind directly to NRP1, and the VEGF_165_/NRP1 complex can subsequently be internalized. Because VEGF_121 _is unable to bind directly to NRP1, this isoform does not undergo the same amount of NRP1-mediated clearance, as compared to VEGF_165_. To better reflect experimental data, we set the isoform secretion ratio VEGF_165_:VEGF_121 _in the tumor to be 50%:50% and use this value in all simulations presented below.

### Free VEGF is predicted to decrease in the normal and tumor compartments and increase in the blood following intravenous administration of an anti-VEGF agent

**Figure 4:**
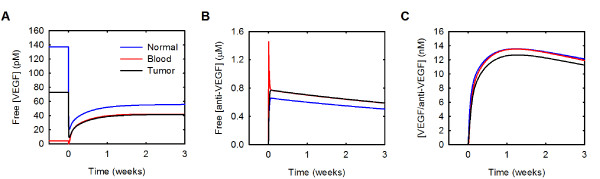
**Whole-body changes in response to anti-VEGF treatment**. Concentration profiles following a single intravenous injection of 10 mg/kg anti-VEGF given at time 0. **A**, VEGF concentration. **B**, Anti-VEGF concentration. **C**, Concentration of the VEGF/anti-VEGF complex.

It has been shown experimentally that VEGF in the blood plasma increases following anti-VEGF treatment [47-50], and our previous model reproduces this increase [21]. The model also predicts that free VEGF in the tumor decreases and remains below baseline following intravenous administration of anti-VEGF. This led to the hypothesis that the anti-VEGF agent acts to deplete VEGF from the tumor, rather than from the blood [21]. Our current model also reproduces the increase in blood plasma free VEGF following anti-VEGF therapy; free VEGF in the blood is predicted to transiently decrease and then increase 9.2-fold three weeks following anti-VEGF treatment (Figure 4A). The current model also supports the hypothesis that a consequence of treatment with an anti-VEGF agent is a depletion of VEGF in the tumor. Following administration of the anti-VEGF, the decrease of free VEGF in the normal and tumor compartments is due to the formation of the VEGF/anti-VEGF complex (Additional file 4). Additionally, less VEGF is bound to its receptors and sequestered in the matrix following treatment.

We have investigated the effects of various model parameters on the distribution of free VEGF, free anti-VEGF, and the VEGF/anti-VEGF complex in the body. Specifically, we evaluated how systemic properties, drug characteristics, and properties of the tumor microenvironment influence the response to VEGF-neutralizing drugs. As a measure of the response to anti-VEGF treatment, we calculated the fold-change in tumor free VEGF at three weeks following administration of the anti-VEGF agent. The three-week time point was selected because free VEGF concentration in the body is not significantly different after multiple cycles where the anti-VEGF is administered every three weeks. Therefore, we simulate one cycle of anti-VEGF treatment and calculate the fold-change as:

(1) $$\text{fold-change}=\frac{{\left[VEGF\right]}_{t=3weeks}}{{\left[VEGF\right]}_{t=0}}$$

The fold-change is a ratio that indicates whether free VEGF increased (fold-change > 1), decreased (fold-change < 1), or was unchanged (fold-change = 1) at a specific time following an anti-VEGF treatment, compared to the steady-state level prior to treatment. We are particularly interested in understanding the conditions for which free VEGF in the tumor decreases following anti-VEGF treatment, which corresponds to a fold-change in tumor VEGF less than one and is termed a *therapeutic effect*. The fold-change in tumor VEGF is 0.6 for the set of parameter values used in the current model.

We have performed a sensitivity study in order to determine the conditions for which the anti-VEGF has a therapeutic effect. The sensitivity study systematically investigates the impact of individual model parameters over the entire simulation period and allows us to quantify the effect of these parameters on model outputs of interest: the fold-change in tumor VEGF and the concentration profiles of VEGF, anti-VEGF, and the VEGF/anti-VEGF complex. We have used the model to predict the fold-change and concentration profiles for a range of parameter values and compare to the model outputs obtained when the initial, baseline value is used. The results of the sensitivity study are presented below. For each model parameter examined, we state the range of values explored.

### Varying systemic properties can alter VEGF distribution throughout the body or in specific compartments and can influence the response to anti-VEGF therapy

**Figure 5:**
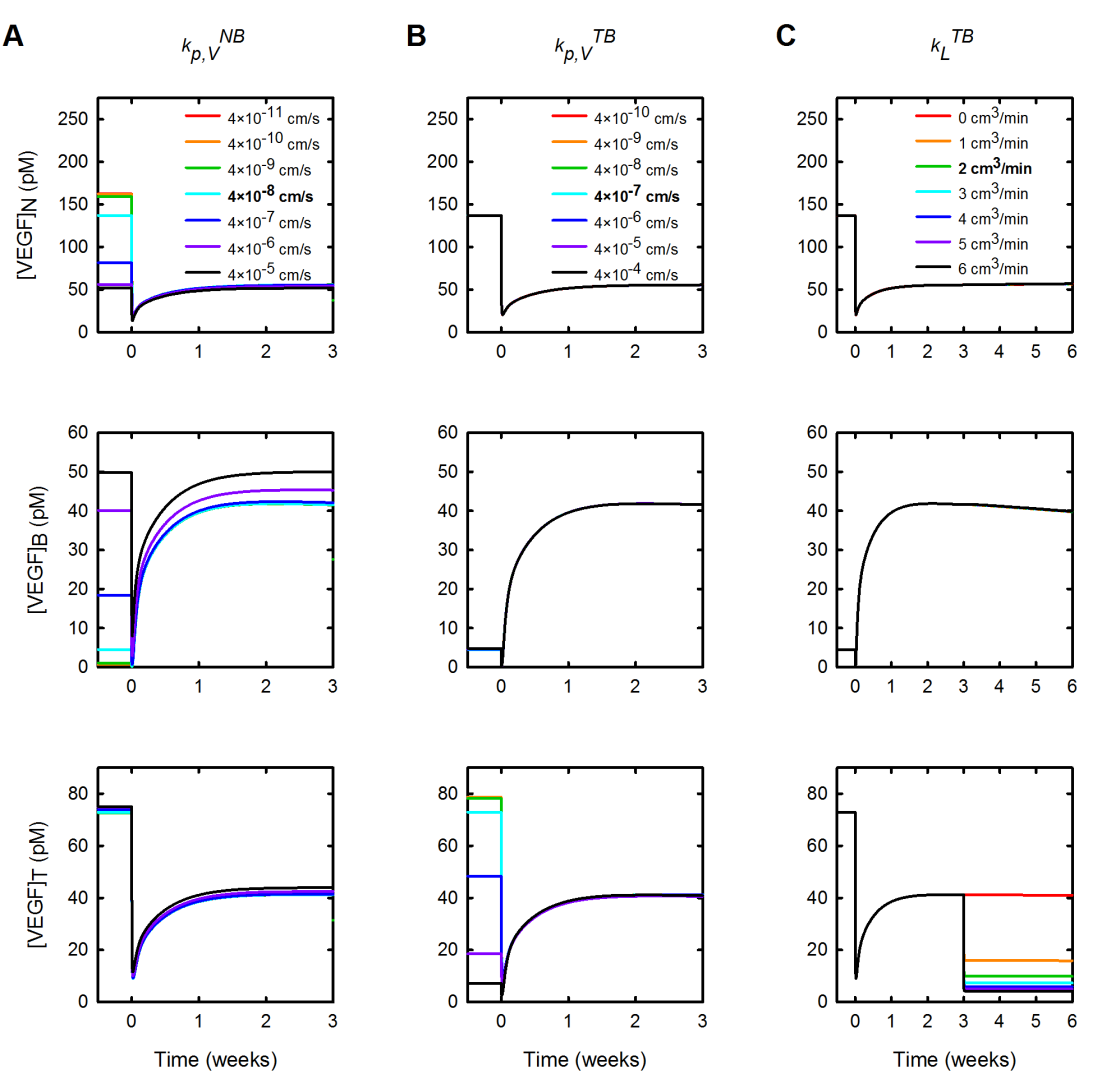
**Effect of systemic properties permeability**. The concentration of free VEGF in the body following anti-VEGF treatment is predicted for various parameter values. **A**, Microvascular permeability to VEGF between the normal tissue and blood. **B**, Microvascular permeability to VEGF between the tumor and blood. **C**, Lymphatic flow from tumor to blood. From top to bottom: normal tissue (subscript *N*), blood (subscript *B*), and tumor (subscript *T*). Bold in the legend indicates parameter value used in the current model.

We examined the impact of varying microvascular permeability between the normal tissue or tumor and the blood ($k_{p,V}^{NB}$ and $k_{p,V}^{TB}$, respectively) and of varying lymphatic drainage (*k_L_*). The microvascular permeability to VEGF between the normal tissue and blood was varied from 4 × 10^-11 ^to 4 × 10^-5 ^cm/s. Increasing $k_{p,V}^{NB}$ had a pronounced effect on the concentration of free VEGF in the normal tissue and plasma prior to the anti-VEGF injection (Figure 5A). As $k_{p,V}^{NB}$ increases, the steady-state concentration of VEGF in the normal tissue decreases prior treatment, and the fold-change in free VEGF concentration becomes approximately one (Figure 5A top panel). In the blood, increasing $k_{p,V}^{NB}$ leads to an increase in the concentration of free VEGF before and after anti-VEGF treatment; however, the fold-change in free VEGF in the blood remains greater than one for all values of $k_{p,V}^{NB}$ examined (Figure 5A middle panel). Varying $k_{p,V}^{NB}$ does not substantially influence free VEGF in the tumor (Figure 5A bottom panel).

The effects of varying microvascular permeability to VEGF between the tumor and blood are confined to the tumor compartment (Figure 5B). We varied $k_{p,V}^{TB}$ from 4 × 10^-10 ^to 4 × 10^-4 ^cm/s. As $k_{p,V}^{TB}$ increases, free VEGF in the normal tissue and blood plasma are relatively unchanged (Figure 5B, top and middle panels). In comparison, upon increasing $k_{p,V}^{TB}$, the concentration of VEGF in the tumor decreases prior to the anti-VEGF injection (Figure 5B, bottom panel). After the injection, tumor free VEGF increases slightly with increasing $k_{p,V}^{TB}$ and there is a therapeutic effect when $k_{p,V}^{TB}$ is less than 10^-5 ^cm/s. These results indicate that making the tumor vasculature more permeable to VEGF impedes the therapeutic response to the anti-VEGF agent in the tumor.

Varying lymphatic drainage in the body does not produce noticeable changes in the VEGF distribution in the body; however, the rate of lymph flow does impact the distribution of free anti-VEGF and the VEGF/anti-VEGF complex (Additional file 5). Increasing *k_L _*results in a decrease in the concentration of free anti-VEGF and the VEGF/anti-VEGF complex in normal tissue, while the opposite trend is observed in the blood plasma and tumor. The model predicts that increasing lymphatic drainage allows the maximum amount of anti-VEGF to be present in the blood and tumor compartments.

Although tumor lymphatics are non-functional, there is evidence that normalization of the tumor vasculature occurs with anti-VEGF treatment, interstitial pressure is reduced, and lymphatics may become functional [51]. Therefore, we also investigated the effect of introducing lymphatic flow from the tumor to the blood three weeks after anti-VEGF treatment (Figure 5C). The effects of allowing lymph drainage from the tumor is confined to that particular compartment and results in a reduction in tumor free VEGF that is distinct from the depletion induced by the anti-VEGF. Thus, the model predicts that if anti-VEGF therapy is able to restore tumor lymph flow, the treatment has an additive effect of further reducing tumor VEGF. Free VEGF in the tumor decreases from 41 pM to 4.2 pM when *k_L _*is varied from 0 to 6 cm^3^/min.

### Properties of the anti-VEGF agent influence the distribution of free VEGF in the body and impact the response to treatment

**Figure 6:**
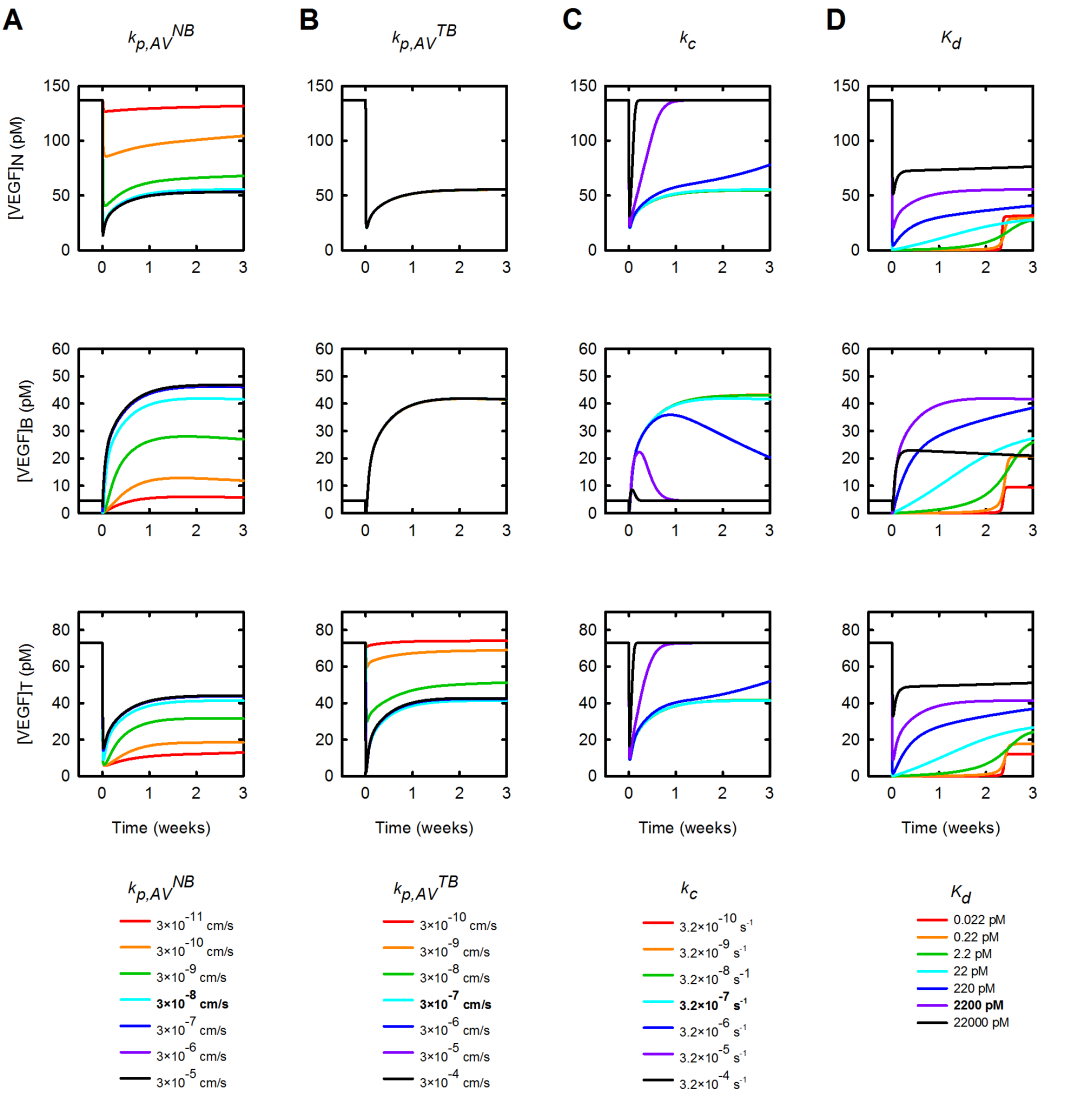
**Effect of anti-VEGF properties**. The concentration of free VEGF in the body following anti-VEGF treatment is predicted as properties of the anti-VEGF are varied. **A**, Effect of microvascular permeability to anti-VEGF between the normal tissue and blood. **B**, Effect of microvascular permeability to anti-VEGF between the tumor and blood. **C**, Effect of clearance rate of anti-VEGF and VEGF/anti-VEGF complex. **D**, Effect of anti-VEGF binding affinity for VEGF. From top to bottom: normal tissue, blood, and tumor. Bold in the legend indicates parameter value used in the current model.

Figure 6 shows how the concentration of free VEGF changes in response to varying the permeability to the anti-VEGF in the normal tissue and tumor (Figures 6A and 6B, respectively), the rate of clearance of anti-VEGF (Figure 6C), and the binding affinity of the anti-VEGF to VEGF (Figure 6D). We varied the microvascular permeability to the anti-VEGF agent and the VEGF/anti-VEGF complex in the normal tissue from 3 × 10^-11 ^to 3 × 10^-5 ^cm/s, which induced changes in the distribution of VEGF throughout the body. Increasing $k_{p,AV}^{NB}$ results in a decrease in VEGF in the normal tissue following anti-VEGF treatment (Figure 6A). Conversely, following treatment, free VEGF in the tumor and blood both increase as $k_{p,AV}^{NB}$ increases. Although tumor free VEGF increases following treatment, the fold-change in free VEGF is less than one for all values of $k_{p,AV}^{NB}$ examined, indicating that the anti-VEGF agent induces the desired therapeutic effect.

The permeability to anti-VEGF in the tumor has an effect on the tumor compartment only (Figure 6B). Increasing $k_{p,AV}^{TB}$ from 3 × 10^-10 ^to 3 × 10^-4 ^cm/s leads to a decrease in free VEGF in the tumor from 74 pM to 43 pM at three weeks post-treatment. A therapeutic effect is observed for the values of $k_{p,AV}^{TB}$ examined, with the exception of $k_{p,AV}^{TB}$ on the order of 10^-10 ^cm/s. In that case, the fold-change is only slightly greater than one.

The clearance of the anti-VEGF and the VEGF/anti-VEGF complex influences the concentrations of these species, as well as VEGF, in all compartments, as shown in Figure 6C. We varied *k_c _*from 3.2 × 10^-10 ^to 3.2 × 10^-4 ^s^-1^, which changes the time it takes for the concentrations of VEGF, anti-VEGF and the complex to return to their steady-state values (see also Additional files 6 and 7). Larger values of *k_c _*cause the concentration of these species to return to their steady-state values more quickly. For example, when *k_c _*is 3.2 × 10^-4 ^s^-1^, tumor VEGF rebounds to its pre-treatment level within 4 days; however, when *k_c _*is 3.2 × 10^-10 ^s^-1^, tumor VEGF goes to a pseudo steady-state and requires several weeks to return to the pre-treatment level. Thus, increasing the clearance rate for the anti-VEGF and VEGF/anti-VEGF complex impedes the therapeutic action of the anti-VEGF agent in the tumor.

The concentrations of VEGF, anti-VEGF, and the VEGF/anti-VEGF complex in the body were sensitive to the binding affinity of the drug to VEGF. Decreasing *K_d _*leads to a decrease in the concentration of free VEGF in the tumor, where tumor VEGF decreases 4.2-fold when *K_d _*is varied from 0.022 pM to 22 nM. (Figure 6D). Interestingly, for *K_d_*<2.2 pM, the concentration of free VEGF in all compartments immediately goes to nearly zero until free anti-VEGF is cleared from the body (Additional file 7), at which point free VEGF rebounds to a new pseudo steady-state level within three weeks of anti-VEGF treatment. The binding affinity also influences free anti-VEGF concentration and has a particularly significant impact on the concentration of the VEGF/anti-VEGF complex (Additional file 6). At *K_d _*< 220 pM, the concentration of the complex in all compartments increases to approximately 600 μM.

**Figure 7:**
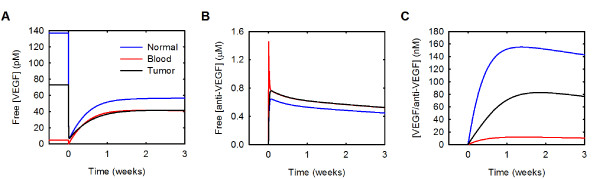
**Effect of anti-VEGF binding matrix-bound VEGF**. Concentration profiles following a single intravenous injection of 10 mg/kg anti-VEGF given at time 0 when the anti-VEGF is able to bind matrix-bound VEGF. **A**, VEGF concentration. **B**, Anti-VEGF concentration. **C**, VEGF/anti-VEGF complex concentration.

**Figure 8:**
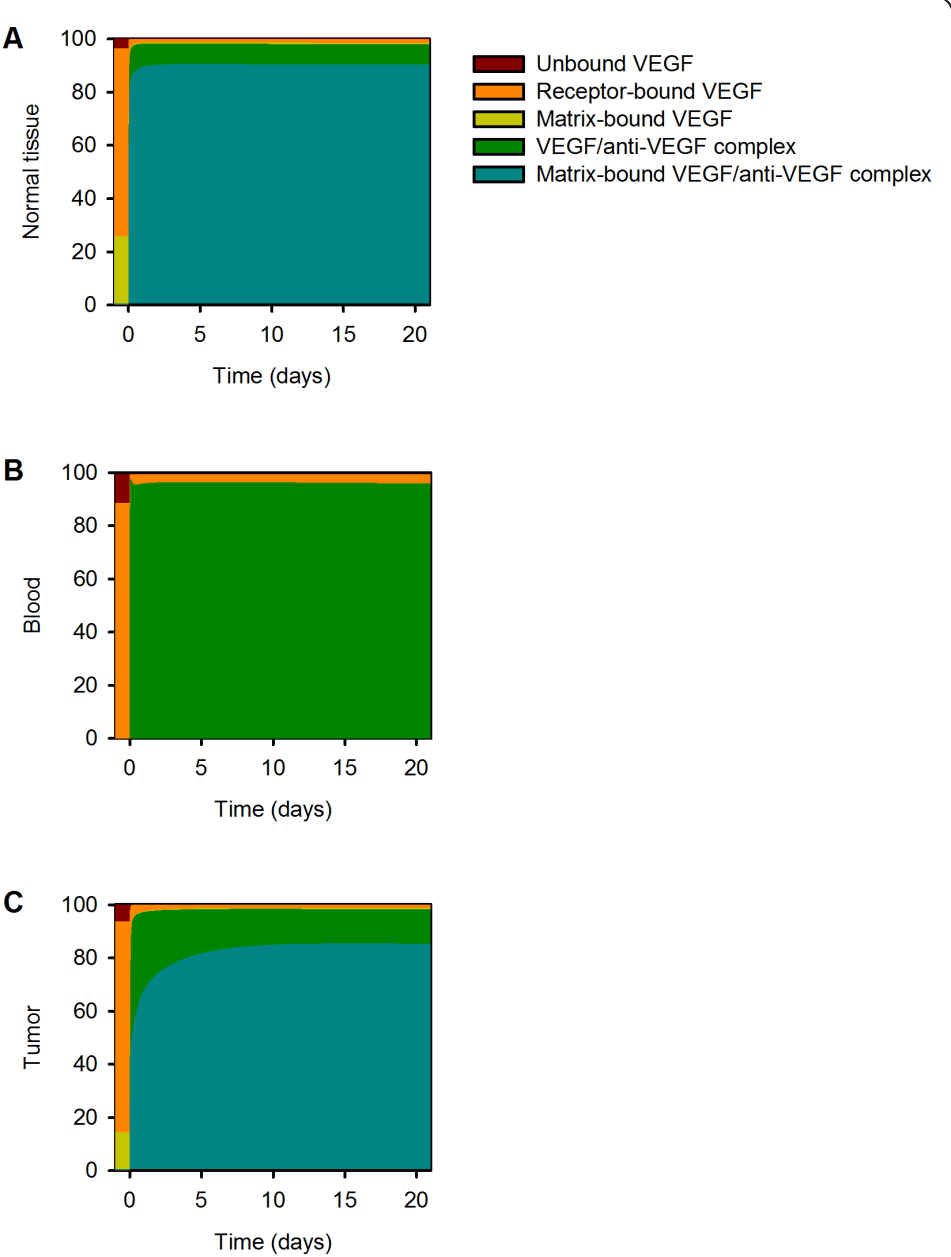
**VEGF distribution with anti-VEGF binding matrix-bound VEGF**. VEGF distribution in the body. **A**, Normal tissue. **B**, Blood. **C**, Tumor.

At steady state, 22% and 13% of total VEGF in the normal tissue and tumor, respectively, is sequestered by the extracellular matrix (Additional file 4); however, it is not known how anti-VEGF binding to matrix-bound VEGF would influence the distribution of VEGF in the body. The VEGF residues important for antibody binding and those responsible for receptor binding occupy a common region [52]. Since the receptor-binding region is distinct from the binding site for GAG chains in the extracellular matrix, it is possible that the anti-VEGF binds to matrix-bound VEGF. Therefore, we investigated the effect of allowing the anti-VEGF to bind to VEGF sequestered in the extracellular matrix. The concentration profiles for VEGF, free anti-VEGF and the VEGF/anti-VEGF complex when anti-VEGF can bind to VEGF sequestered in the ECM are illustrated in Figure 7. Allowing the anti-VEGF to bind to matrix-bound VEGF influences the time required for VEGF concentration to reach a pseudo-steady state after treatment; however, the fold-change in free VEGF at three weeks post-treatment is unaffected. The ability of the anti-VEGF to bind matrix-bound VEGF does not therefore alter the predicted therapeutic effect. Approximately 90% of VEGF is bound to both the anti-VEGF and the ECM following anti-VEGF treatment (Figure 8). As a result, the concentration of the VEGF/anti-VEGF complex in the interstitial space of the normal tissue and tumor is 13- and 7-fold larger, respectively, when anti-VEGF binds matrix-bound VEGF (Figure 7 compared to Figure 4, and Figure 8 compared to Additional file 4).

**Figure 9:**
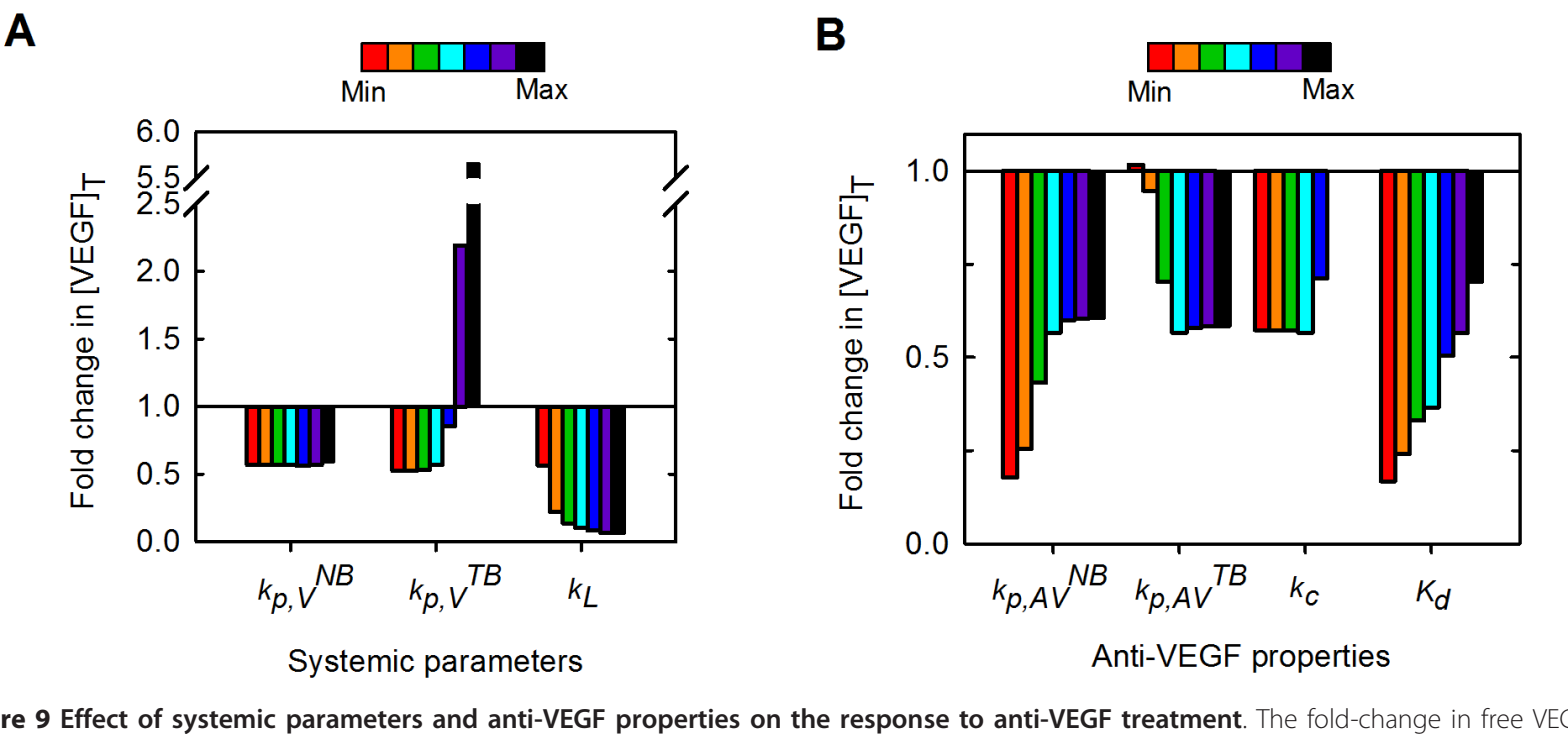
**Effect of systemic parameters and anti-VEGF properties on the response to anti-VEGF treatment**. The fold-change in free VEGF concentration following anti-VEGF treatment as a function of various model parameters. **A**, Systemic parameters. **B**, Anti-VEGF properties. Color bar indicates range of values for each parameter, as given in Figures 5 and 6.

The effects of the systemic parameters and anti-VEGF properties on the fold-change in free VEGF in the tumor are summarized in Figure 9. As we have seen, our model predicts how substantial changes in systemic parameters and in pharmacokinetic and pharmacodynamic parameters of the anti-VEGF agent influence the concentration of free VEGF in the tumor following administration of the anti-VEGF agent. Free VEGF in the tumor is predicted to decrease following anti-VEGF treatment for all of the parameters and ranges of parameter values examined in this study, with the exception of $k_{p,V}^{TB}$, the microvascular permeability to VEGF between the tumor and blood. In this case, the model predicts that when $k_{p,V}^{TB}$ is on the order of 10^-5 ^cm/s or larger, free VEGF in the tumor increases following anti-VEGF therapy. Thus, the therapeutic effect of the anti-VEGF is robust and occurs for a wide range of values for systemic parameters and drug properties.

### Tumor-specific characteristics influence the response to anti-VEGF treatment

**Figure 10:**
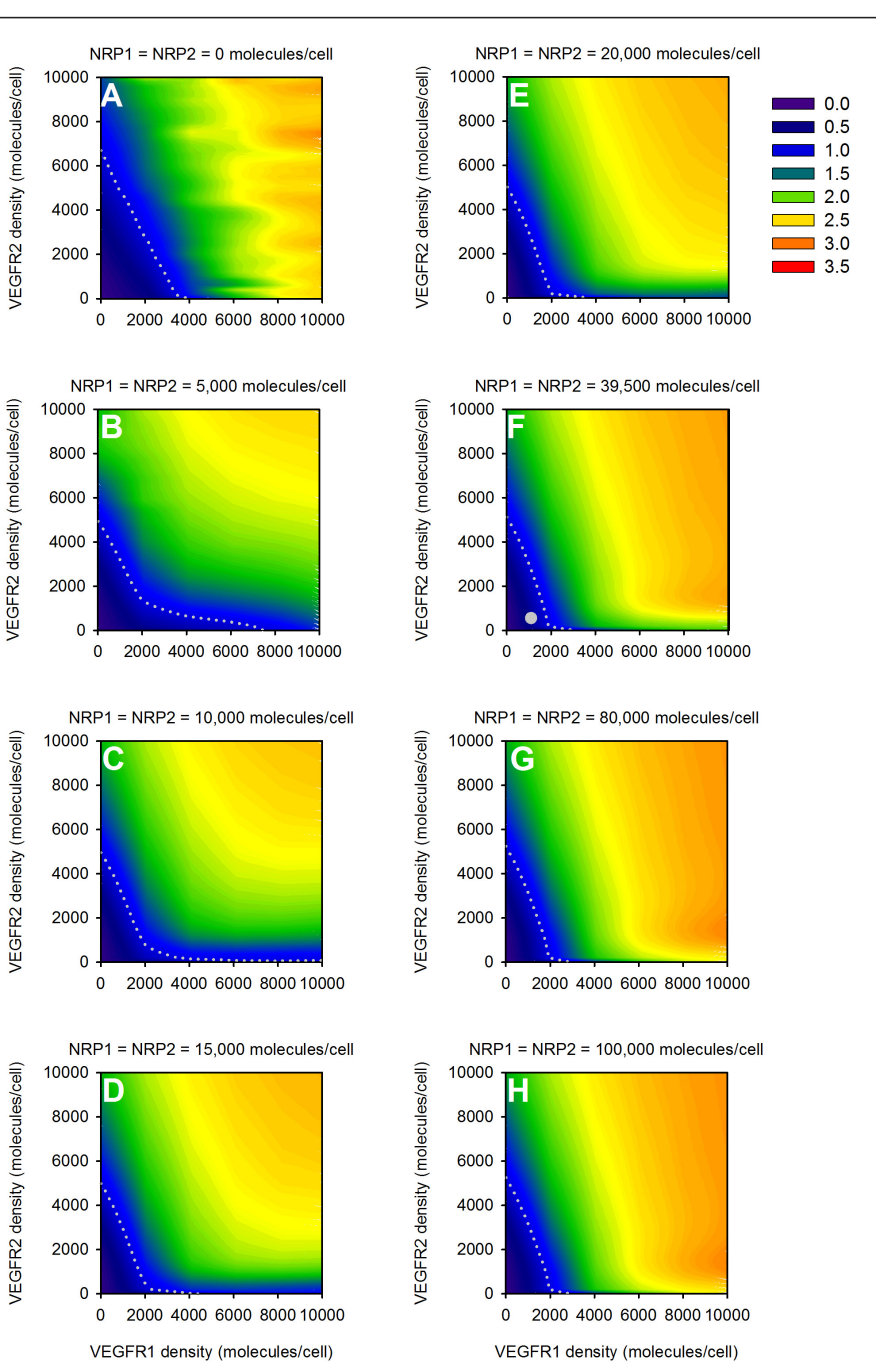
**Effect of receptor density on tumor cells**. The fold-change in free VEGF concentration following anti-VEGF treatment is predicted as a function of VEGFR1, VEGFR2, and neuropilin (NRP) expression. In all simulations, NRP1 = NRP2. **A**, NRP = 0 molecules/tumor cell. **B**, NRP = 5,000 molecules/tumor cell. **C**, NRP = 10,000 molecules/tumor cell. **D**, NRP = 15,000 molecules/tumor cell. **E**, NRP = 20,000 molecules/tumor cell. **F**, NRP = 39,500 molecules/tumor cell. The white circle indicates receptor densities used in the current model: VEGFR1 = 1,100 molecules/tumor cell, VEGFR2 = 550 molecules/tumor cell, NRP1 = NRP2 = 39,500 molecules/tumor cell. **G**, NRP = 80,000 molecules/tumor cell. **H**, NRP = 100,000 molecules/tumor cell. **A-H**, The gray dotted line in all panels is the isocline for a fold-change of 1.

We examined the role of the tumor microenvironment and how tumor-specific characteristics influence the response to anti-VEGF therapy. We first investigated the effect of VEGF receptor expression on tumor cells. The densities of VEGR1 and VEGFR2 were varied from 0 to 10,000 receptors/tumor cell, and the densities of NRP1 and NRP2 were varied from 0 to 100,000 receptors/tumor cell. This study reveals the dependence of the concentration of free VEGF in the body and the response to anti-VEGF treatment on receptor expression on tumor cells (Figure 10). There are combinations of VEGF receptor densities for which tumor VEGF is predicted to decrease following intravenous injection of the anti-VEGF agent, revealing the parameter space where anti-VEGF treatment has a therapeutic effect. The fold-change of tumor free VEGF in response to the treatment is sensitive to expression of VEGF receptors and co-receptors. The threshold value for VEGFR1 expression needed to result in a therapeutic effect depends on neuropilin expression. As the expression of neuropilins increases, the level of VEGFR1 required to result in a fold-change less than one in tumor free VEGF decreases. The threshold value for the density of VEGFR2 required to obtain a fold-change less than one is approximately 7,000 receptors/tumor cell. The instance where there are no NRPs on tumor cells is an exceptional case, and the threshold values for VEGFR1 and VEGFR2 are 10,000 and 6,000 receptors/tumor cell, respectively.

**Figure 11:**
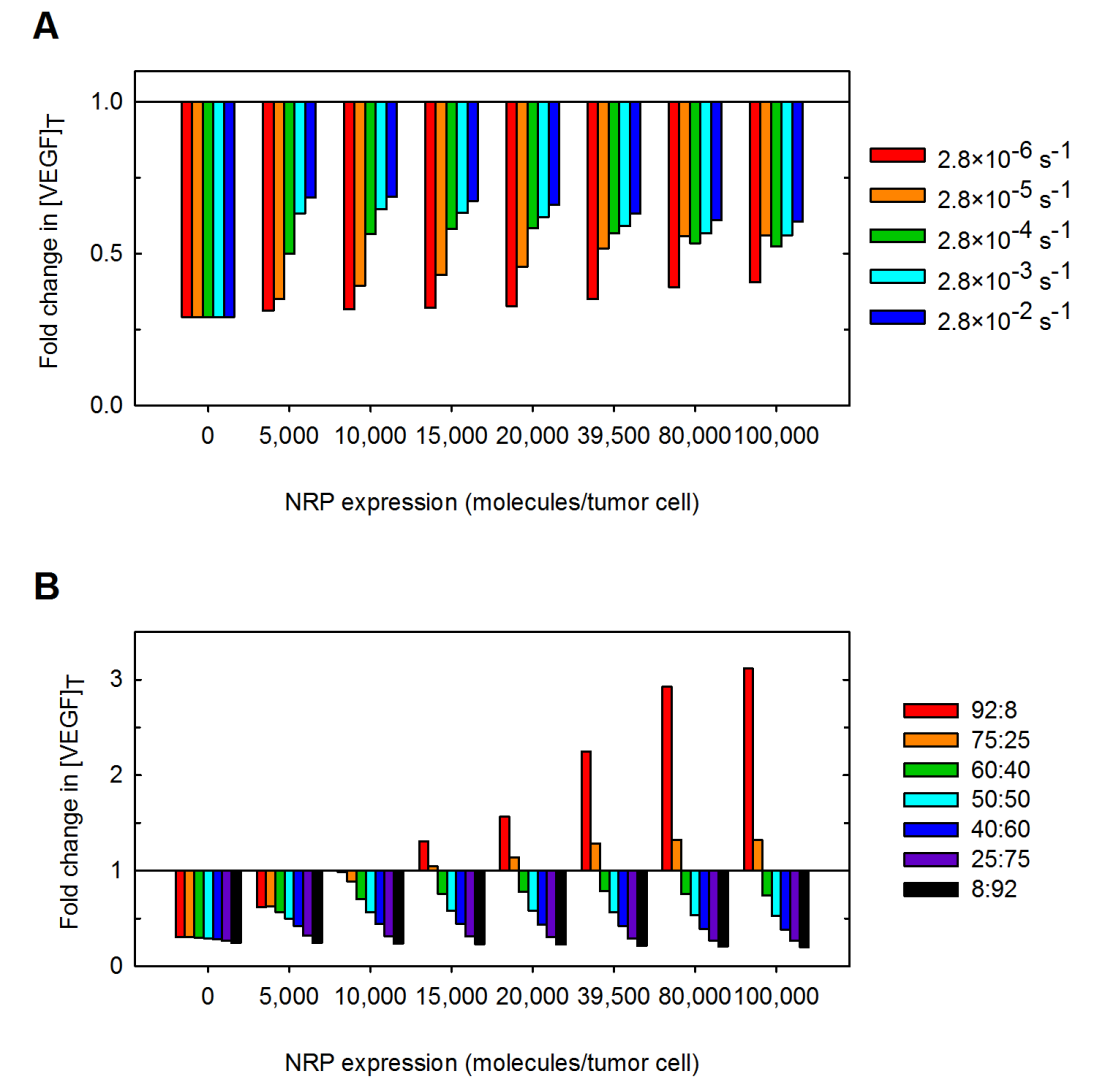
**Effect of tumor microenvironment on the response to the anti-VEGF treatment**. The fold-change in free VEGF concentration following anti-VEGF treatment is predicted as a function of properties of the tumor microenvironment. **A**, Effect of neuropilin internalization. **B**, Effect of VEGF isoform-specific secretion.

We next examined the rate of internalization of neuropilin co-receptors to determine its effect on the response to anti-VEGF therapy (Figure 11A). Neuropilins greatly outnumber VEGFR1 and VEGFR2 in the tumor and can form ternary complexes with VEGF and VEGFR1 or VEGFR2. For these reasons, the availability of neuropilins on the cell surface alters the dynamics of anti-VEGF treatment. The model predicts that as the rate of internalization of neuropilins increases from 2.8 × 10^-6 ^to 2.8 × 10^-2 ^s^-1^, the fold-change in free VEGF in the tumor increases for neuropilin densities up to 80,000 molecules/tumor cell. There is a complex relationship between NRP internalization and the response to anti-VEGF treatment when neuropilin density exceeds about 80,000 molecules/tumor cell. In general, as the internalization rate of neuropilins increases, the therapeutic effect decreases, which is due to the complex interactions between VEGF, neuropilins, and the anti-VEGF. Decreasing NRP internalization leads to a decrease in the rate at which VEGF is internalized due to being bound to NRP1 or NRP2. As a result, VEGF now has time to unbind from NRPs and subsequently be sequestered by the anti-VEGF agent. These results demonstrate that the anti-VEGF agent has an increased therapeutic effect as the rate of NRP internalization decreases.

Lastly, we examined the effect of VEGF secretion in the tumor. As we show above, the isoform secretion ratio influences the steady-state concentration of free VEGF in the body (Figure 3B). Therefore, we sought to understand the impact of the expression of VEGF isoforms on the response to anti-VEGF treatment. The isoform secretion ratio VEGF_165_:VEGF_121 _in the tumor was varied between 100%:0% to 0%:100% from its current value of 50:50%, while keeping the total VEGF secretion rate constant. The isoform secretion ratio in normal tissue remained at 92%:8%. As tumor VEGF secretion includes more VEGF_121_, the fold-change decreases to less than one (Figure 11B). Thus, a therapeutic effect is observed when VEGF_121 _comprises at least 25% of the total VEGF secreted from the tumor cells.

## Discussion

We have developed a molecularly-detailed model of VEGF transport in the body. This is the first human model to include experimental measurements of receptor densities as well as receptors on parenchymal cells, and thus represents a significant advance compared to previous models. By incorporating *in vivo *and *in vitro *quantifications of VEGF receptor expression on endothelial and parenchymal cells and experimentally-based values for VEGF degradation in the tissue compartments and VEGF secretion by tumors, we have created a predictive tool that reflects physiological elements of VEGF-mediated angiogenesis. Using the model, we are able to predict how systemic properties, drug design parameters, and properties of the tumor microenvironment influence the response to anti-VEGF treatment. Specifically, we have predicted the fold-change in free VEGF following intravenous injection of an anti-VEGF agent. Importantly, the model predicted that the outcome of the anti-VEGF treatment (e.g., the level of free VEGF in the tumor interstitium) is dependent on the tumor microenvironment (e.g., receptor expression, internalization of neuropilins, and VEGF isoform ratio) and therefore may vary from patient to patient or between groups of patients. The model predicted that transport and binding parameters of the anti-VEGF can be fine-tuned such that the drug acts to deplete free VEGF in the tumor.

Our model predicted that tight binding between the anti-VEGF and VEGF results in a strong therapeutic effect. Although free VEGF was reduced to nearly zero in all compartments in the few hours following the treatment, and the desired therapeutic effect is observed, tight binding between VEGF and the anti-VEGF agent may also induce adverse effects. The work of Gerber *et al. *[53] shows a positive correlation between tight binding of the anti-VEGF to VEGF and the inhibition of tumor growth; however, tighter association between anti-VEGF and VEGF results in increased toxicity. Specifically, the authors identify renal changes that result from increased antibody affinity, including glomerulosclerosis and anti-VEGF deposition in glomeruli, as well as hypoalbuminemia and ascites formation [53]. Further investigation into the mechanism of glomerular injury reveals that inhibition of VEGF in noncancerous tissues such as the kidney results in a reduction of glomerular VEGF, which is required for the health and integrity of the adjacent microvasculature [54]. As VEGF is required for cell maintenance and tissue homeostasis, inhibiting endogenous VEGF signaling leads to additional side effects [55]. Thus, there is an optimal value for the binding affinity where the anti-VEGF binds VEGF tightly enough to inhibit tumor growth while limiting toxicity, and this drug design parameter must be carefully balanced with the occurrence of adverse events.

The occurrence of the desired therapeutic effect is sensitive to the expression of VEGF receptors on tumor cells. Our current model incorporates *in vivo *experimental data for the density of VEGFR1 and VEGFR2 located on the normal and tumor endothelia, muscle fibers (myocytes), and tumor cells. In contrast, the number of neuropilin receptors on various cell types has yet to be quantified *in vivo*. Our analysis shows that the occurrence of a therapeutic effect is dependent on the density of VEGFRs and co-receptors. Therefore, accurate estimates of neuropilin expression and the relative density of NRP1 compared to NRP2 are required, as the response to anti-VEGF therapy is sensitive to this property of the tumor microenvironment.

In addition to the density of neuropilin expression on tumor cells, the availability of these receptors also influences the response to anti-VEGF treatment. Since neuropilins are co-receptors involved in angiogenesis and are estimated to be present in large numbers on parenchymal cells, it is possible that the internalization of these receptors may be dysregulated in pathological conditions. Our model predicts that a low rate of internalization of neuropilins leads to a more drastic reduction of free VEGF in the tumor. A previous model was used to compare therapeutic approaches of targeting NRP1 and shows that blocking NRP1 expression does not result in persistent inhibition of VEGF signaling [56]. Our results support this finding, and in fact show that prolonged expression of NRPs (inhibiting NRP internalization) will improve the therapeutic effect of anti-VEGF treatment. Teesalu and coworkers recently characterized the amino acid motif of NRP1 that promotes its internalization and found that blocking interaction at that site inhibits internalization of the receptor [57]. Their work suggests that it is possible to specifically target neuropilin internalization.

The rate at which VEGF is secreted and the VEGF isoform secretion ratio VEGF_165_:VEGF_121 _in the tumor have a significant impact on the response to anti-VEGF treatment and are crucial for prediction of the therapeutic effect. Specifically, the rate of VEGF secretion in the tumor can be used to tune the steady-state level of free VEGF in the tumor and influences whether an anti-VEGF agent works to deplete tumor VEGF. Our model predicts that the steady-state concentration of tumor free VEGF prior to treatment influences whether the anti-VEGF has a therapeutic effect. This underscores the need for isolation of tumor interstitial fluid and measurement of its VEGF concentration, which may be used as a predictive biomarker for administration of specific anti-angiogenic drugs and serve in stratification of patients who would best respond to a specific therapy.

Similarly, there is a need for quantitative measurements of the relative secretion of VEGF isoforms. These data would aid in refining the model and would lead to a better understanding of the effects of drugs that target VEGF-mediated angiogenesis. It is interesting to note that the VEGF isoform ratio in tumor tissues is in the range required for an anti-VEGF agent to have a therapeutic effect, as compared to the ratio observed in other tissues such as muscle. These results bring into question whether the VEGF isoform expression may be a useful biomarker to predict a therapeutic response to anti-VEGF treatment.

We have shown that the properties of the tumor should be taken into account when developing treatment strategies. It would be of interest to predict the effect of anti-VEGF treatment when using receptor quantification and the relative secretion of VEGF isoforms for specific types of tumors. This type of analysis may provide insight as to why certain tumors respond to anti-VEGF treatment better than others. Additionally, the incorporation of tumor-specific properties is required to develop personalized medicine and identify the patient population that is best-suited for VEGF-targeted therapies [58].

Our model of VEGF distribution is used to investigate the effect of anti-VEGF agents in targeting VEGF and inhibiting angiogenesis. An alternative or complementary view is that the anti-VEGF therapy works through vascular normalization, i.e., repairing tumor vasculature to resemble normal vessels, leading to increased pericyte coverage and increased blood perfusion, reduced interstitial pressure, and tightened endothelial cell junctions [51]. Clinically, metastasis is reduced and the efficacy of chemo-, radiation- and immune-therapies is improved upon vessel normalization [7]. Interestingly, our model predicts that when tumor lymphatics become functional, perhaps due to a reduction in interstitial pressure following normalization, the anti-VEGF has a more potent therapeutic effect. Similarly, it would be of interest to incorporate the dynamic effects of vascular normalization on macromolecular permeability, which is reduced following normalization, in order to understand how this influences the response to anti-VEGF therapy. Vascular normalization is a transient response, characterized by an optimal time window after which the normalized features of the tumor vasculature are lost, possibly due to prolonged anti-VEGF treatment or development of a resistance to treatment [7,51,59]. Our current model predicts that tumor VEGF is immediately reduced following anti-VEGF treatment and then rebounds to a new pseudo-steady state below pre-treatment levels within 7 days. The time it takes for tumor VEGF to rebound may correspond to the normalization window. Therefore, our model can be used to explore how the duration of the normalization window depends on systemic and drug parameters and tumor properties.

Several assumptions influence the model predictions and should be re-evaluated as quantitative experimental data become available. We have assumed equal distribution of receptors on the luminal and abluminal surface of endothelial cells. Previous work predicts that quantification of luminal and abluminal receptors influences VEGF distribution in the body [34]. We have assumed that the total number of receptors is conserved; however receptor expression is a dynamic process and the cell-surface receptor density might depend on VEGF concentration among other factors [31]. Additionally, the administration of an anti-VEGF agent may affect receptor expression over days and weeks, the time scale investigated in the present study. Therefore, incorporating receptor dynamics and quantifying the effect of anti-VEGF therapy on receptor density would better reflect biological conditions. Anti-VEGF therapies influence the tumor vasculature and may also have anti-tumor effects leading to tumor growth inhibition. However, we have assumed that the size of the tumor remains constant throughout the model simulation. It would be of interest to incorporate a function for tumor growth and/or regression as a function of time. Lastly, the effects of platelet content or the ability of platelets to secrete and sequester angiogenic proteins has not been addressed. Since degranulation of platelets is a source of VEGF and platelets have been shown to sequester the VEGF antibody bevacizumab [60], it is important to add platelets to the model.

## Conclusions

The model predicted that the therapeutic response to anti-VEGF treatment is robust across a large range of systemic and drug parameter values. However, properties of the tumor microenvironment such as receptor expression and availability and VEGF isoform secretion ratio significantly influence the response to anti-VEGF therapy. Our results are important in elucidating effects of drug design and tumor-specific parameters that are difficult to predict *a priori *and may be helpful in optimizing VEGF-neutralizing drugs.

## Authors' contributions

SDF, MOS, and ASP conceived the study. SDF performed the simulations and wrote the manuscript. PII performed the experiments. All authors participated in the analysis of the results and read and approved the final manuscript.

## Supplementary Material

Additional file 1 **Chemical reactions and equations for the compartment model of VEGF distribution in the body**. This file also contains a glossary of terms used in the model equations. Click here for file

Additional file 2 **We investigated how the kinetic parameters governing NRP2 binding to VEGF_165 _and coupling to VEGFR1 and VEGFR2 influenced the steady-state concentration of free VEGF in the body**. **A**, Effect of VEGF_165 _binding to NRP2; squares and triangles indicate lower and upper values of *k_on_*, respectively, taken from literature. **B**, Effect of NRP2 coupling to VEGF_165_/VEGFR2. **C**, Effect of VEGF_165_/NRP2 coupling to VEGFR2. D, Effect of NRP2 coupling to VEGFR1. In all panels, gray circles indicate baseline values taken from NRP1 interactions. Click here for file

Additional file 3 **Comparison of the concentration of free VEGF in the tumor interstitial space calculated from experimental data and based on model predictions**. Click here for file

Additional file 4 **VEGF distribution following a single intravenous injection of 10 mg/kg of anti-VEGF given at time 0**. **A**, normal tissue, **B**, blood, and **C**, tumor. Click here for file

Additional file 5 **The concentration profiles for A, free VEGF; B, free anti-VEGF; and C, the VEGF/anti-VEGF complex are predicted as the lymphatic flow rate from the normal tissue to the blood was varied**. From top to bottom: normal tissue, blood, and tumor. The lymph flow rate influences the concentration of the anti-VEGF and VEGF/anti-VEGF complex. Legend in A applies to all panels; bold in the legend indicates the parameter value used in the current model. Click here for file

Additional file 6 **The concentration of the VEGF/anti-VEGF complex in the body following anti-VEGF treatment is predicted as properties of the anti-VEGF are varied**. **A**, Effect of microvascular permeability to anti-VEGF between the normal tissue and blood. **B**, Effect of microvascular permeability to anti-VEGF between the tumor and blood. **C**, Effect of clearance rate of anti-VEGF. **D**, Effect of anti-VEGF binding affinity to VEGF. From top to bottom: normal tissue, blood, and tumor. Bold in the legend indicates parameter value used in the current model. Click here for file

Additional file 7 **The concentration of free anti-VEGF in the body following anti-VEGF treatment is predicted as properties of the anti-VEGF are varied**. **A**, Effect of microvascular permeability to anti-VEGF between the normal tissue and blood. **B**, Effect of microvascular permeability to anti-VEGF between the tumor and blood. **C**, Effect of clearance rate of anti-VEGF. **D**, Effect of anti-VEGF binding affinity to VEGF. From top to bottom: normal tissue, blood, and tumor. Bold in the legend indicates parameter value used in the current model. Click here for file
