# Supplementary material for: Pharmacokinetics and pharmacodynamics of VEGF-neutralizing antibodies
Source: BMC Syst Biol. 2011 Nov 21;5:193. doi: 10.1186/1752-0509-5-193 (PMC3229549; doi:10.1186/1752-0509-5-193)
Supplement: Additional file 1 — Chemical reactions and equations for the compartment model of VEGF distribution in the body. This file also contains a glossary of terms used in the model equations. [file 1752-0509-5-193-S1.DOC]

Chemical reactions

The relevant chemical reactions are presented here (molecular species and parameters are defined in the glossary):

Equations

The complete list of ordinary differential equations is presented below:

1. **Interstitial space in normal tissue compartment**

$\begin{matrix} \frac{d[V_{165}]_{N}}{dt}=q_{V165}^{N}-k_{on,V165,MEBM}^{N}[V_{165}]_{N}[M_{EBM}]_{N}+k_{off,V165,MEBM}^{N}[V_{165}M_{EBM}]_{N} \\ -k_{on,V165,MPBM}^{N}[V_{165}]_{N}[M_{PBM}]_{N}+k_{off,V165,MPBM}^{N}[V_{165}M_{PBM}]_{N} \\ -k_{on,V165,MECM}^{N}[V_{165}]_{N}[M_{ECM}]_{N}+k_{off,V165,MECM}^{N}[V_{165}M_{ECM}]_{N} \\ -k_{on,V165,R1}^{N}[V_{165}]_{N}[R_{1}]_{N}+k_{off,V165R1}^{N}[V_{165}R_{1}]_{N} \\ -k_{on,V165,R2}^{N}[V_{165}]_{N}[R_{2}]_{N}+k_{off,V165R2}^{N}[V_{165}R_{2}]_{N} \\ -k_{on,V165,N1}^{N}[V_{165}]_{N}[N_{1}]_{N}+k_{off,V164N1}^{N}[V_{165}N_{1}]_{N} \\ -k_{on,V165,N1}^{N,myo}[V_{165}]_{N}[N_{1}]_{N,myo}+k_{off,V164N1}^{N,myo}[V_{165}N_{1}]_{N,myo} \\ -k_{on,V165,A}^{N}[V_{165}]_{N}[A]_{N}+k_{off,V164A}^{N}[V_{165}A]_{N} \\ -\left(\frac{k_{L}+k_{pV}^{NB}S_{NB}}{U_{N}}\right)\frac{[V_{165}]_{N}}{K_{AV,N}}+k_{pV}^{BN}\frac{S_{NB}}{U_{N}}\frac{U_{B}}{U_{P}}[V_{165}]_{B} \end{matrix}$ (S.1)

$\begin{matrix} \frac{d[V_{121}]_{N}}{dt}=q_{V121}^{N}-k_{on,V121,R1}^{N}[V_{121}]_{N}[R_{1}]_{N}+k_{off,V121R1}^{N}[V_{121}R_{1}]_{N} \\ -k_{on,V121,R1N1}^{N}[V_{121}]_{N}[R_{1}N_{1}]_{N}+k_{off,V121R1N1}^{N}[V_{121}R_{1}N_{1}]_{N} \\ -k_{on,V121,R2}^{N}[V_{12}]_{N}[R_{2}]_{N}+k_{off,V121R2}^{N}[V_{121}R_{2}]_{N} \\ -k_{on,V121,A}^{N}[V_{121}]_{N}[A]_{N}+k_{off,V121A}^{N}[V_{121}A]_{N} \\ -\left(\frac{k_{L}+k_{pV}^{NB}S_{NB}}{U_{N}}\right)\frac{[V_{121}]_{N}}{K_{AV,N}}+k_{pV}^{BN}\frac{S_{NB}}{U_{N}}\frac{U_{B}}{U_{P}}[V_{121}]_{B} \end{matrix}$ (S.2)

$\frac{d[M_{EBM}]_{N}}{dt}=-k_{on,V165,MEBM}^{N}[V_{165}]_{N}[M_{EBM}]_{N}+k_{off,V165MEBM}^{N}[V_{165}M_{EBM}]_{N}$ (S.3)

$\frac{d[M_{PBM}]_{N}}{dt}=-k_{on,V165,MPBM}^{N}[V_{165}]_{N}[M_{PBM}]_{N}+k_{off,V165MPBM}^{N}[V_{165}M_{PBM}]_{N}$ (S.4)

$\frac{d[M_{ECM}]_{N}}{dt}=-k_{on,V165,MECM}^{N}[V_{165}]_{N}[M_{ECM}]_{N}+k_{off,V165MECM}^{N}[V_{165}M_{ECM}]_{N}$ (S.5)

$\frac{d[V_{165}M_{EBM}]_{N}}{dt}=k_{on,V165,MEBM}^{N}[V_{165}]_{N}[M_{EBM}]_{N}-k_{off,V165MEBM}^{N}[V_{165}M_{EBM}]_{N}$ (S.6)

$\frac{d[V_{165}M_{PBM}]_{N}}{dt}=k_{on,V165,MPBM}^{N}[V_{165}]_{N}[M_{PBM}]_{N}-k_{off,V165MPBM}^{N}[V_{165}M_{PBM}]_{N}$ (S.7)

$\frac{d[V_{165}M_{ECM}]_{N}}{dt}=k_{on,V165,MECM}^{N}[V_{165}]_{N}[M_{ECM}]_{N}-k_{off,V165MECM}^{N}[V_{165}M_{ECM}]_{N}$ (S.8)

$\begin{matrix} \frac{d[R_{1}]_{N}}{dt}=s_{R1}^{N}-k_{int,R1}^{N}[R_{1}]_{N}-k_{on,V165,R1}^{N}[V_{165}]_{N}[R_{1}]_{N}+k_{off,V165R1}^{N}[V_{165}R_{1}]_{N} \\ -k_{on,V121,R1}^{N}[V_{121}]_{N}[R_{1}]_{N}+k_{off,V121R1}^{N}[V_{121}R_{1}]_{N} \\ -k_{c,R1,N1}^{N}[N_{1}]_{N}[R_{1}]_{N}+k_{dissoc,R1N1}^{N}[R_{1}N_{1}]_{N} \end{matrix}$ (S.9)

$\begin{matrix} \frac{d[R_{2}]_{N}}{dt}=s_{R2}^{N}-k_{int,R2}^{N}[R_{2}]_{N}-k_{on,V121,R2}^{N}[V_{121}]_{N}[R_{2}]_{N}+k_{off,V120R2}^{N}[V_{121}R_{2}]_{N} \\ -k_{on,V1645R2}^{N}[V_{165}]_{N}[R_{2}]_{N}+k_{off,V165R2}^{N}[V_{165}R_{2}]_{N} \\ -k_{c,V165N1,R2}^{N}[V_{165}N_{1}]_{N}[R_{2}]_{N}+k_{off,V165N1,R2}^{N}[R_{2}V_{165}N_{1}]_{N} \end{matrix}$ (S.10)

$\begin{matrix} \frac{d[N_{1}]_{N}}{dt}=s_{N1}^{N}-k_{int,N1}^{N}[N_{1}]_{N}-k_{c,V121R1,N1}^{N}[V_{121}R_{1}]_{N}[N_{1}]_{N}+k_{dissoc,R1N1}^{N}[V_{121}R_{1}N_{1}]_{N} \\ -k_{c,R1,N1}^{N}[N_{1}]_{N}[R_{1}]_{N}+k_{dissoc,R1N1}^{N}[R_{1}N_{1}]_{N} \\ -k_{on,V165,N1}^{N}[V_{165}]_{N}[N_{1}]_{N}+k_{off,V165N1}^{N}[V_{165}N_{1}]_{N} \\ -k_{c,V165R2,N1}^{N}[V_{165}R_{2}]_{N}[N_{1}]_{N}+k_{off,V165R2,N1}^{N}[R_{2}V_{165}N_{1}]_{N} \end{matrix}$ (S.11)

$\begin{matrix} \frac{d[N_{1}]_{N,myo}}{dt}=s_{N1}^{N,myo}-k_{\mathrm{int},N1}^{N,myo}[N_{1}]_{N,myo} \\ +k_{on,V165,N1}^{N,myo}[V_{165}]_{N}[N_{1}]_{N,myo}-k_{off,V165N1}^{N,myo}[V_{165}N_{1}]_{N,myo} \end{matrix}$ (S.12)

$\frac{d[V_{165}R_{1}]_{N}}{dt}=-k_{int,V165R1}^{N}[V_{164}R_{1}]_{N}+k_{on,V165,R1}^{N}[V_{165}]_{N}[R_{1}]_{N}-k_{off,V165R1}^{N}[V_{165}R_{1}]_{N}$ (S.13)

$\begin{matrix} \frac{d[V_{165}R_{2}]_{N}}{dt}=-k_{int,V165R2}^{N}[V_{165}R_{2}]_{N}+k_{on,V165,R2}^{N}[V_{165}]_{N}[R_{2}]_{N}-k_{off,V165R2}^{N}[V_{165}R_{2}]_{N} \\ -k_{c,V165R2,N1}^{N}[V_{165}R_{2}]_{N}[N_{1}]_{N}+k_{off,V165R2N1}^{N}[R_{2}V_{165}N_{1}]_{N} \end{matrix}$ (S.14)

$\begin{matrix} \frac{d[V_{165}N_{1}]_{N}}{dt}=-k_{int,V165N1}^{N}[V_{165}N_{1}]_{N}+k_{on,V165,N1}^{N}[V_{165}]_{N}[N_{1}]_{N}-k_{off,V165N1}^{N}[V_{165}N_{1}]_{N} \\ -k_{c,V165N1,R2}^{N}[V_{165}N_{1}]_{N}[R_{2}]_{N}+k_{off,V165N1R2}^{N}[R_{2}V_{165}N_{1}]_{N} \end{matrix}$ (S.15)

$\begin{matrix} \frac{d[R_{2}V_{165}N_{1}]_{N}}{dt}=-k_{int,V165R2N1}^{N}[R_{2}V_{165}N_{1}]_{N} \\ +k_{c,V165R2,N1}^{N}[V_{165}R_{2}]_{N}[N_{1}]_{N}-k_{off,V165R2N1}^{N}[R_{2}V_{165}N_{1}]_{N} \\ +k_{c,V165N1,R2}^{N}[V_{165}N_{1}]_{N}[R_{2}]_{N}-k_{off,V165N1R2}^{N}[R_{2}V_{165}N_{1}]_{N} \end{matrix}$ (S.16)

$\begin{matrix} \frac{d[V_{121}R_{1}]_{N}}{dt}=-k_{int,V121R1}^{N}[V_{121}R_{1}]_{N} \\ +k_{on,V121,R1}^{N}[V_{121}]_{N}[R_{1}]_{N}-k_{off,V121R1}^{N}[V_{121}R_{1}]_{N} \\ -k_{c,R1,N1}^{N}[V_{121}R_{1}]_{N}[N_{1}]_{N}+k_{dissoc,R1N1}^{N}[V_{121}R_{1}N_{1}]_{N} \end{matrix}$ (S.17)

$\frac{d[V_{121}R_{2}]_{N}}{dt}=-k_{int,V121R2}^{N}[V_{121}R_{2}]_{N}+k_{on,V121,R2}^{N}[V_{121}]_{N}[R_{2}]_{N}-k_{off,V121R2}^{N}[V_{121}R_{2}]_{N }$ (S.18)

$\begin{matrix} \frac{d[R_{1}N_{1}]_{N}}{dt}=-k_{int,R1N1}^{N}[R_{1}N_{1}]_{N} \\ +k_{c,R1,N1}^{N}[R_{1}]_{N}[N_{1}]_{N}-k_{dissoc,R1N1}^{N}[R_{1}N_{1}]_{N} \\ -k_{on,V121,R1}^{N}[V_{121}]_{N}[R_{1}N_{1}]_{N}+k_{off,V121R1}^{N}[V_{121}R_{1}N_{1}]_{N} \end{matrix}$ (S.19)

$\begin{matrix} \frac{d[V_{121}R_{1}N_{1}]_{N}}{dt}=-k_{intV121R1N1}^{N}[V_{121}R_{1}N_{1}]_{N} \\ +k_{c,V121R1,N1}^{N}[V_{121}R_{1}]_{N}[N_{1}]_{N}-k_{dissoc,V121N1}^{N}[V_{121}R_{1}N_{1}]_{N} \\ +k_{on,V121R1N1}^{N}[V_{121}]_{N}[R_{1}N_{1}]_{N}-k_{off,V121R1N1}^{N}[V_{121}R_{1}N_{1}]_{N} \end{matrix}$ (S.20)

$\begin{matrix} \frac{d[V_{165}N_{1}]_{N.myo}}{dt}=-k_{int,V165N1}^{N,myo}[V_{165}N_{1}]_{N,myo} \\ +k_{on,V165,N1}^{N,myo}[V_{165}]_{N}[N_{1}]_{N,myo}-k_{off,V165N1}^{N,myo}[V_{165}N_{1}]_{N,myo} \end{matrix}$ (S.21)

$\begin{matrix} \frac{d[A]_{N}}{dt}=-k_{on,V165,A}^{N}[V_{165}]_{N}[A]_{N}+k_{off,V165A}^{N}[V_{165}A]_{N} \\ -k_{on,V121,A}^{N}[V_{121}]_{N}[A]_{N}+k_{off,V121A}^{N}[V_{121}A]_{N} \\ +k_{pA}^{BN}\frac{S_{NB}}{U_{N}}\frac{U_{B}}{U_{P}}[A]_{B}-\left(\frac{k_{L}+k_{pA}^{NB}S_{NB}}{U_{N}}\right)\frac{[A]_{N}}{K_{AV,N}} \end{matrix}$ (S.22)

$\begin{matrix} \frac{d[V_{165}A]_{N}}{dt}=k_{on,V165,A}^{N}[V_{165}]_{N}[A]_{N}-k_{off,V165A}^{N}[V_{165}A]_{N} \\ +k_{pA}^{BN}\frac{S_{NB}}{U_{N}}\frac{U_{B}}{U_{p}}[V_{165}A]_{B}-\left(\frac{k_{L}+k_{pA}^{NB}S_{NB}}{U_{N}}\right)\frac{[V_{165}A]_{N}}{K_{AV,N}} \end{matrix}$ (S.23)

$\begin{matrix} \frac{d[V_{121}A]_{N}}{dt}=k_{on,V121,A}^{N}[V_{121}]_{N}[A]_{N}-k_{off,V121A}^{N}[V_{121}A]_{N} \\ +k_{pA}^{BN}\frac{S_{NB}}{U_{N}}\frac{U_{B}}{U_{p}}[V_{121}A]_{B}-\left(\frac{k_{L}+k_{pA}^{NB}S_{NB}}{U_{N}}\right)\frac{[V_{121}A]_{N}}{K_{AV,N}} \end{matrix}$ (S.24)

1. **Interstitial space in blood compartment**

We denote the luminal receptors and ligand-receptor complexes on endothelial cells (ECs) by the subscript *i* (*i=N* for normal ECs; *i=T* for diseased ECs).

$\begin{matrix} \frac{d[V_{165}]_{B}}{dt}=-c_{V165}[V_{165}]_{B}-k_{on,V165,R1}^{B}[V_{165}]_{B}[R_{1}]_{B,i}+k_{off,V165R1}^{B}[V_{165}R_{1}]_{B,i} \\ -k_{on,V165,R2}^{B}[V_{165}]_{B}[R_{2}]_{B,i}+k_{off,V165R2}^{B}[V_{165}R_{2}]_{B,i} \\ -k_{on,V165,N1}^{B}[V_{165}]_{B}[N_{1}]_{B,i}+k_{off,V165N1}^{B}[V_{165}N_{1}]_{B,i} \\ -k_{on,V165,A}^{B}[V_{165}]_{B}[A]_{B}+k_{off,V165A}^{B}[V_{165}A]_{B} \\ -\frac{k_{pV}^{BN}S_{NB}}{U_{p}}[V_{165}]_{B}+\left(\frac{k_{L}+k_{pV}^{NB}S_{NB}}{U_{B}}\right)\frac{[V_{165}]_{N}}{K_{AV,N}} \end{matrix}$ (S.25)

$\begin{matrix} \frac{d[V_{121}]_{B}}{dt}=-c_{V121}[V_{121}]_{B}-k_{on,V121,R1}^{B}[V_{121}]_{B}[R_{1}]_{B,i}+k_{off,V121R1}^{B}[V_{121}R_{1}]_{B,i} \\ -k_{on,V121,R1N1}^{B}[V_{121}]_{B}[R_{1}N_{1}]_{B,i}+k_{off,V121R1N1}^{B}[V_{121}R_{1}N_{1}]_{B,i} \\ -k_{on,V121,R2}^{B}[V_{121}]_{B}[R_{2}]_{B,i}+k_{off,V121R2}^{B}[V_{121}R_{2}]_{B,i} \\ -k_{on,V121,A}^{B}[V_{121}]_{B}[A]_{B}+k_{off,V121A}^{B}[V_{121}A]_{B} \\ -\frac{k_{pV}^{BN}S_{NB}}{U_{p}}[V_{121}]_{B}+\left(\frac{k_{L}+k_{pV}^{NB}S_{NB}}{U_{B}}\right)\frac{[V_{121}]_{N}}{K_{AV,N}} \end{matrix}$ (S.26)

$\begin{matrix} \frac{d[R_{1}]_{B,i}}{dt}=s_{R1}^{B}-k_{int,R1}^{B}[R_{1}]_{B,i}-k_{on,V165,R1}^{B} [V_{164}]_{B}[R_{1}]_{B,i}+k_{off,V165R1}^{B}[V_{165}R_{1}]_{B,i} \\ -k_{on,V121,R1}^{B}[V_{121}]_{B}[R_{1}]_{B,i}+k_{off,V121R1}^{B}[V_{121}R_{1}]_{B,i} \\ -k_{c,R1,N1}^{B}[N_{1}]_{B,i}[R_{1}]_{B,i}+k_{dissoc,R1N1}^{B}[R_{N}N_{1}]_{B,i} \end{matrix}$ (S.27)

$\begin{matrix} \frac{d[R_{2}]_{B,i}}{dt}=s_{R2}^{B}-k_{int,R2}^{B} [R_{2}]_{B,i}-k_{on,V121,R2}^{B}[V_{121}]_{B}[R_{2}]_{B,i}+k_{off,V121R2}^{B}[V_{121}R_{2}]_{B,i} \\ -k_{on,V165,R2}^{B}[V_{165}]_{B}[R_{2}]_{B,i}+k_{off,V165R2}^{B}[V_{165}R_{2}]_{B,i} \\ -k_{c,V165N1,R2}^{B}[V_{165}N_{1}]_{B}[R_{2}]_{B,i}+k_{off,V165N1,R2}^{B}[R_{2}V_{165}N_{1}]_{B,i} \end{matrix}$ (S.28)

$\begin{matrix} \frac{d[N_{1}]_{B,i}}{dt}=s_{N1}^{B}-k_{int,N1}^{B}[N_{1}]_{B,i}-k_{c,V121R1,N1}^{B}[V_{121}R_{1}]_{B}[N_{1}]_{B,i}+k_{dissoc,R1N1}^{B}[V_{121}R_{1}N_{1}]_{B,i} \\ -k_{c,R1,N1}^{B}[N_{1}]_{B,i}[R_{1}]_{B,i}+k_{dissoc,R1N1}^{B}[R_{1}N_{1}]_{B,i} \\ -k_{on,V165,N1}^{B}[V_{165}]_{B}[N_{1}]_{B,i}+k_{off,V165N1}^{B}[V_{165}N_{1}]_{B,i} \\ -k_{c,V165R2,N1}^{B}[V_{165}R_{2}]_{B,i}[N_{1}]_{B,i}+k_{off,V165R2,N1}^{B}[R_{2}V_{165}N_{1}]_{B,i} \end{matrix}$ (S.29)

$\frac{d[V_{165}R_{1}]_{B,i}}{dt}=-k_{int,V165R1}^{B}[V_{165}R_{1}]_{B,i}+k_{on,V165,R1}^{B}[V_{165}]_{B}[R_{1}]_{B,i}-k_{off,V165R1}^{B}[V_{165}R_{1}]_{B,i}$ (S.30)

$\frac{d[V_{165}R_{2}]_{B,i}}{dt}=-k_{int,V165R2}^{B}[V_{165}R_{2}]_{B,i}+k_{on,V165,R2}^{B}[V_{165}]_{B}[R_{2}]_{B,i}-k_{off,V165R2}^{B}[V_{165}R_{2}]_{B,i}$ (S.31)

$\begin{matrix} \frac{d[V_{165}N_{1}]_{B,i}}{dt}=-k_{int,V165N1}^{B}[V_{165}N_{1}]_{B,i}+k_{on,V165,N1}^{B}[V_{165}]_{B}[N_{1}]_{B,i}-k_{off,V165N1}^{B}[V_{165}N_{1}]_{B,i} \\ -k_{c,V165N1,R2}^{B}[V_{165}N_{1}]_{B,i}[R_{2}]_{B,i}+k_{off,V165N1R2}^{B}[R_{2}V_{165}N_{1}]_{B,i} \end{matrix}$ (S.32)

$\begin{matrix} \frac{d[R_{2}V_{165}N_{1}]_{B,i}}{dt}=-k_{int,V165R2N1}^{B}[R_{2}V_{165}N_{1}]_{B,i} \\ +k_{c,V165R2,N1}^{B}[V_{165}R_{2}]_{B,i}[N_{1}]_{B,i}-k_{off,V165R2N1}^{B}[R_{2}V_{165}N_{1}]_{B,i} \\ +k_{c,V165N1,R2}^{B}[V_{165}N_{1}]_{B,i}[R_{2}]_{B,i}-k_{off,V165N1R2}^{B}[R_{2}V_{165}N_{1}]_{B,i} \end{matrix}$ (S.33)

$\begin{matrix} \frac{d[V_{121}R_{1}]_{B,i}}{dt}=-k_{int,V121R1}^{B}[V_{121}R_{1}]_{B,i} \\ +k_{on,V121,R1}^{B}[V_{121}]_{B,i}[R_{1}]_{B,i}-k_{off,V121R1}^{B}[V_{121}R_{1}]_{B,i} \\ -k_{c,R1,N1}^{B}[V_{121}R_{1}]_{B,i}[N_{1}]_{B,i}+k_{dissoc,R1N1}^{B}[V_{121}R_{1}N_{1}]_{B,i} \end{matrix}$ (S.34)

$\frac{d[V_{121}R_{2}]_{B,i}}{dt}=-k_{int,V121R2}^{B}[V_{121}R_{2}]_{B,i}+k_{on,V121,R2}^{B}[V_{121}]_{B}[R_{2}]_{B,i}-k_{off,V121R2}^{B}[V_{121}R_{2}]_{B,i}$ (S.35)

$\begin{matrix} \frac{d[R_{1}N_{1}]_{B,i}}{dt}=-k_{int,R1N1}^{B}[R_{1}N_{1}]_{B,i} \\ +k_{c,R1,N1}^{B}[R_{1}]_{B,i}[N_{1}]_{B,i}-k_{dissoc,R1N1}^{B}[R_{1}N_{1}]_{B,i} \\ -k_{on,V121,R1}^{B}[V_{121}]_{B}[R_{1}N_{1}]_{B,i}+k_{off,V121R1}^{B}[V_{121}R_{1}N_{1}]_{B,i} \end{matrix}$ (S.36)

$\begin{matrix} \frac{d[V_{121}R_{1}N_{1}]_{B,i}}{dt}=-k_{intV121R1N1}^{B}[V_{121}R_{1}N_{1}]_{B,i}\text{ } \\ +k_{c,V121R1,N1}^{B}[V_{121}R_{1}]_{B,i}[N_{1}]_{B,i}-k_{dissoc,V121N1}^{B}[V_{121}R_{1}N_{1}]_{B,i} \\ +k_{on,V121R1N1}^{B}[V_{121}]_{B}[R_{1}N_{1}]_{B,i}-k_{off,V121R1N1}^{B}[V_{121}R_{1}N_{1}]_{B,i} \end{matrix}$ (S.37)

$\begin{matrix} \frac{d[A]_{B}}{dt}=q_{A}-c_{A}[A]_{B}-k_{on,V165,A}^{B}[V_{165}]_{B}[A]_{B}+k_{off,V165A}^{B}[V_{165}A]_{B} \\ -k_{on,V121,A}^{B}[V_{121}]_{B}[A]_{B}+k_{off,V121A}^{B}[V_{121}A]_{B} \\ +k_{pA}^{BN}\frac{S_{NB}}{U_{p}}[A]_{B}-\left(\frac{k_{L}+k_{pA}^{NB}S_{NB}}{U_{B}}\right)\frac{[A]_{N}}{K_{AV,N}} \end{matrix}$ (S.38)

$\begin{matrix} \frac{d[V_{165}A]_{B}}{dt}=-c_{V165A}[V_{165}A]_{B}+k_{on,V165,A}^{B}[V_{165}]_{B}[A]_{B}-k_{off,V165A}^{B}[V_{165}A]_{B} \\ -k_{pA}^{BN}\frac{S_{NB}}{U_{p}}[V_{165}A]_{B}+\left(\frac{k_{L}+k_{pA}^{NB}S_{NB}}{U_{B}}\right)\frac{[V_{165}A]_{N}}{K_{AV,N}} \end{matrix}$ (S.39)

$\begin{matrix} \frac{d[V_{121}A]_{B}}{dt}=-c_{V121A}[V_{121}A]_{B}+k_{on,V121,A}^{B}[V_{121}]_{B}[A]_{B}-k_{off,V121A}^{B}[V_{121}A]_{B} \\ -k_{pA}^{BN}\frac{S_{NB}}{U_{p}}[V_{121}A]_{B}+\left(\frac{k_{L}+k_{pA}^{NB}S_{NB}}{U_{B}}\right)\frac{[V_{121}A]_{N}}{K_{AV,N}} \end{matrix}$ (S.40)

1. **Interstitial space in tumor compartment**

We denote the receptors and ligand-receptor complexes by the subscript *i* (*i=*T for tumor ECs; *i=tumor* for tumor cells).

$\begin{matrix} \frac{d[V_{165}]_{T}}{dt}=q_{V165}^{T}-k_{on,V165,MEBM}^{T}[V_{165}]_{T}[M_{EBM}]_{T}+k_{off,V165,MEBM}^{T}[V_{165}M_{EBM}]_{T} \\ -k_{on,V165,MPBM}^{T}[V_{165}]_{T}[M_{PBM}]_{T}+k_{off,V165,MPBM}^{T}[V_{165}M_{PBM}]_{T} \\ -k_{on,V165,MECM}^{T}[V_{165}]_{T}[M_{ECM}]_{T}+k_{off,V165,MECM}^{T}[V_{165}M_{ECM}]_{T} \\ -k_{on,V165,R1}^{T,i}[V_{165}]_{T}[R_{1}]_{T,i}+k_{off,V165R1}^{T}[V_{165}R_{1}]_{T,i} \\ -k_{on,V165,R2}^{T,i}[V_{165}]_{T}[R_{2}]_{T,i}+k_{off,V165R2}^{T}[V_{165}R_{2}]_{T,i} \\ -k_{on,V165,N1}^{T,i}[V_{165}]_{T}[N_{1}]_{T,i}+k_{off,V164N1}^{T}[V_{165}N_{1}]_{T,i} \\ -k_{on,V165,N2}^{T,tumor}[V_{165}]_{T}[N_{2}]_{T,tumor}+k_{off,V164N2}^{T,tumor}[V_{165}N_{2}]_{T,tumor} \\ -k_{on,V165,A}^{T}[V_{165}]_{T}[A]_{T}+k_{off,V164A}^{T}[V_{165}A]_{T} \\ -\left(\frac{k_{L}+k_{pV}^{TB}S_{TB}}{U_{T}}\right)\frac{[V_{165}]_{T}}{K_{AV,T}}+k_{pV}^{BT}\frac{S_{TB}}{U_{T}}\frac{U_{B}}{U_{P}}[V_{165}]_{B} \end{matrix}$ (S.41)

$\begin{matrix} \frac{d[V_{121}]_{T}}{dt}=q_{V121}^{T}-k_{on,V121,R1}^{T}[V_{121}]_{N}[R_{1}]_{N,i}+k_{off,V121R1}^{T}[V_{121}R_{1}]_{T,i} \\ -k_{on,V121,R1N1}^{T}[V_{121}]_{T}[R_{1}N_{1}]_{T,i}+k_{off,V121R1N1}^{T}[V_{121}R_{1}N_{1}]_{T,i} \\ -k_{on,V121,R2}^{T}[V_{12}]_{T}[R_{2}]_{T,i}+k_{off,V121R2}^{T}[V_{121}R_{2}]_{T,i} \\ -k_{on,V121,A}^{T}[V_{121}]_{T}[A]_{T}+k_{off,V121A}^{T}[V_{121}A]_{T} \\ -\left(\frac{k_{L}+k_{pV}^{TB}S_{TB}}{U_{T}}\right)\frac{[V_{121}]_{T}}{K_{AV,T}}+k_{pV}^{BT}\frac{S_{TB}}{U_{T}}\frac{U_{B}}{U_{P}}[V_{121}]_{B} \end{matrix}$ (S.42)

$\frac{d[M_{EBM}]_{T}}{dt}=-k_{on,V165,MEBM}^{N}[V_{165}]_{T}[M_{EBM}]_{T}+k_{off,V165MEBM}^{T}[V_{165}M_{EBM}]_{T}$ (S.43)

$\frac{d[M_{PBM}]_{T}}{dt}=-k_{on,V165,MPBM}^{T}[V_{165}]_{T}[M_{PBM}]_{T}+k_{off,V165MPBM}^{T}[V_{165}M_{PBM}]_{T}$ (S.44)

$\frac{d[M_{ECM}]_{T}}{dt}=-k_{on,V165,MECM}^{T}[V_{165}]_{T}[M_{ECM}]_{T}+k_{off,V165MECM}^{T}[V_{165}M_{ECM}]_{T}$ (S.45)

$\frac{d[V_{165}M_{EBM}]_{T}}{dt}=k_{on,V165,MEBM}^{T}[V_{165}]_{T}[M_{EBM}]_{T}-k_{off,V165MEBM}^{T}[V_{165}M_{EBM}]_{T}$ (S.46)

$\frac{d[V_{165}M_{PBM}]_{T}}{dt}=k_{on,V165,MPBM}^{T}[V_{165}]_{T}[M_{PBM}]_{T}-k_{off,V165MPBM}^{T}[V_{165}M_{PBM}]_{T}$ (S.47)

$\frac{d[V_{165}M_{ECM}]_{T}}{dt}=k_{on,V165,MECM}^{T}[V_{165}]_{T}[M_{ECM}]_{T}-k_{off,V165MECM}^{T}[V_{165}M_{ECM}]_{T}$ (S.48)

$\begin{matrix} \frac{d[R_{1}]_{T,i}}{dt}=s_{R1}^{T}-k_{int,R1}^{T}[R_{1}]_{T,i}-k_{on,V165,R1}^{T}[V_{165}]_{T}[R_{1}]_{T,i}+k_{off,V165R1}^{T}[V_{165}R_{1}]_{T,i} \\ -k_{on,V121,R1}^{T}[V_{121}]_{T}[R_{1}]_{T,i}+k_{off,V121R1}^{T}[V_{121}R_{1}]_{T,i} \\ -k_{c,R1,N1}^{T}[N_{1}]_{T,i}[R_{1}]_{T,i}+k_{dissoc,R1N1}^{T}[R_{1}N_{1}]_{T,i} \end{matrix}$ (S.49)

$\begin{matrix} \frac{d[R_{2}]_{T,i}}{dt}=s_{R2}^{T}-k_{int,R2}^{T}[R_{2}]_{T,i}-k_{on,V121,R2}^{T}[V_{121}]_{T}[R_{2}]_{T,i}+k_{off,V120R2}^{T}[V_{121}R_{2}]_{T,i} \\ -k_{on,V1645R2}^{T}[V_{165}]_{T}[R_{2}]_{T,i}+k_{off,V165R2}^{T}[V_{165}R_{2}]_{T,i} \\ -k_{c,V165N1,R2}^{T}[V_{165}N_{1}]_{T,i}[R_{2}]_{T,i}+k_{off,V165N1,R2}^{T}[R_{2}V_{165}N_{1}]_{T,i} \end{matrix}$ (S.50)

$\begin{matrix} \frac{d[N_{1}]_{T,i}}{dt}=s_{N1}^{T}-k_{int,N1}^{T}[N_{1}]_{T}-k_{c,V121R1,N1}^{T}[V_{121}R_{1}]_{T,i}[N_{1}]_{T,i}+k_{dissoc,R1N1}^{T}[V_{121}R_{1}N_{1}]_{T,i} \\ -k_{c,R1,N1}^{T}[N_{1}]_{T,i}[R_{1}]_{T,i}+k_{dissoc,R1N1}^{T}[R_{1}N_{1}]_{T,i} \\ -k_{on,V165,N1}^{T}[V_{165}]_{T}[N_{1}]_{T,i}+k_{off,V165N1}^{T}[V_{165}N_{1}]_{T,i} \\ -k_{c,V165R2,N1}^{T}[V_{165}R_{2}]_{T,i}[N_{1}]_{T,i}+k_{off,V165R2,N1}^{T}[R_{2}V_{165}N_{1}]_{T,i} \end{matrix}$ (S.51)

$\begin{matrix} \frac{d[N_{2}]_{T}}{dt}=s_{N2}-k_{\mathrm{int},N2}[N_{2}]_{T} \\ +k_{on,V165,N2}[V_{165}]_{T}[N_{2}]_{T}-k_{off,V165N2}[V_{165}N_{2}]_{T} \end{matrix}$ (S.52)

$\frac{d[V_{165}R_{1}]_{N}}{dt}=-k_{int,V165R1}^{N}[V_{164}R_{1}]_{N}+k_{on,V165,R1}^{N}[V_{165}]_{N}[R_{1}]_{N}-k_{off,V165R1}^{N}[V_{165}R_{1}]_{N}$ (S.53)

$\begin{matrix} \frac{d[V_{165}R_{2}]_{T,i}}{dt}=-k_{int,V165R2}^{T}[V_{165}R_{2}]_{T,i}+k_{on,V165,R2}^{T}[V_{165}]_{T}[R_{2}]_{T,i}-k_{off,V165R2}^{T}[V_{165}R_{2}]_{T,i} \\ -k_{c,V165R2,N1}^{T}[V_{165}R_{2}]_{T,i}[N_{1}]_{T,i}+k_{off,V165R2N1}^{T}[R_{2}V_{165}N_{1}]_{T,i} \end{matrix}$ (S.54)

$\begin{matrix} \frac{d[V_{165}N_{1}]_{T,i}}{dt}=-k_{int,V165N1}^{T}[V_{165}N_{1}]_{T,i}+k_{on,V165,N1}^{T}[V_{165}]_{T}[N_{1}]_{T,i}-k_{off,V165N1}^{T}[V_{165}N_{1}]_{T,i} \\ -k_{c,V165N1,R2}^{T}[V_{165}N_{1}]_{T,i}[R_{2}]_{T,i}+k_{off,V165N1R2}^{T}[R_{2}V_{165}N_{1}]_{T,i} \end{matrix}$ (S.55)

$\begin{matrix} \frac{d[R_{2}V_{165}N_{1}]_{T,i}}{dt}=-k_{int,V165R2N1}^{T}[R_{2}V_{165}N_{1}]_{T,i} \\ +k_{c,V165R2,N1}^{T}[V_{165}R_{2}]_{T,i}[N_{1}]_{T,i}-k_{off,V165R2N1}^{T}[R_{2}V_{165}N_{1}]_{T,i} \\ +k_{c,V165N1,R2}^{T}[V_{165}N_{1}]_{T,i}[R_{2}]_{T,i}-k_{off,V165N1R2}^{T}[R_{2}V_{165}N_{1}]_{T,i} \end{matrix}$ (S.56)

$\begin{matrix} \frac{d[V_{121}R_{1}]_{T,i}}{dt}=-k_{int,V121R1}^{T}[V_{121}R_{1}]_{T,i} \\ +k_{on,V121,R1}^{T}[V_{121}]_{T}[R_{1}]_{T,i}-k_{off,V121R1}^{T}[V_{121}R_{1}]_{T,i} \\ -k_{c,R1,N1}^{T}[V_{121}R_{1}]_{T,i}[N_{1}]_{T,i}+k_{dissoc,R1N1}^{T}[V_{121}R_{1}N_{1}]_{T,i} \end{matrix}$ (S.57)

$\frac{d[V_{121}R_{2}]_{T,i}}{dt}=-k_{int,V121R2}^{T}[V_{121}R_{2}]_{T,i}+k_{on,V121,R2}^{T}[V_{121}]_{T}[R_{2}]_{T,i}-k_{off,V121R2}^{T}[V_{121}R_{2}]_{T,i }$ (S.58)

$\begin{matrix} \frac{d[V_{121}R_{1}N_{1}]_{T}}{dt}=-k_{intV121R1N1}^{T}[V_{121}R_{1}N_{1}]_{T,i} \\ +k_{c,V121R1,N1}^{T}[V_{121}R_{1}]_{T,i}[N_{1}]_{T,i}-k_{dissoc,V121N1}^{T}[V_{121}R_{1}N_{1}]_{T,i} \\ +k_{on,V121R1N1}^{T}[V_{121}]_{T}[R_{1}N_{1}]_{T,i}-k_{off,V121R1N1}^{T}[V_{121}R_{1}N_{1}]_{T,i} \end{matrix}$ (S.59)

$\begin{matrix} \frac{d[R_{1}N_{1}]_{T,i}}{dt}=-k_{int,R1N1}^{T}[R_{1}N_{1}]_{T,i} \\ +k_{c,R1,N1}^{T}[R_{1}]_{T,i}[N_{1}]_{T,i}-k_{dissoc,R1N1}^{T}[R_{1}N_{1}]_{T,i} \\ -k_{on,V121,R1}^{T}[V_{121}]_{T}[R_{1}N_{1}]_{T,i}+k_{off,V121R1}^{T}[V_{121}R_{1}N_{1}]_{T,i} \end{matrix}$ (S.60)

$\begin{matrix} \frac{d[V_{165}N_{2}]_{T}}{dt}=-k_{int,V165N2}^{T}[V_{165}N_{2}]_{T}+k_{on,V165,N2}^{T}[V_{165}]_{T}[N_{2}]_{T}-k_{off,V165N2}^{T}[V_{165}N_{2}]_{T} \\ -k_{c,V165N2,R2}^{T}[V_{165}N_{2}]_{T}[R_{2}]_{T,i}+k_{off,V165N2R2}^{T}[R_{2}V_{165}N_{2}]_{T} \end{matrix}$ (S.61)

$\begin{matrix} \frac{d[R_{2}V_{165}N_{2}]_{T}}{dt}=-k_{int,V165R2N2}^{T}[R_{2}V_{165}N_{2}]_{T} \\ +k_{c,V165R2,N2}^{T}[V_{165}R_{2}]_{T,i}[N_{2}]_{T}-k_{off,V165R2N2}^{T}[R_{2}V_{165}N_{2}]_{T} \\ +k_{c,V165N2,R2}^{T}[V_{165}N_{2}]_{T}[R_{2}]_{T,i}-k_{off,V165N2R2}^{T}[R_{2}V_{165}N_{2}]_{T} \end{matrix}$ (S.62)

$\begin{matrix} \frac{d[R_{1}N_{2}]_{T}}{dt}=-k_{int,R1N1}^{T}[R_{1}N_{2}]_{T} \\ +k_{c,R1,N2}^{T}[R_{1}]_{T,i}[N_{2}]_{T}-k_{dissoc,R1N2}^{T}[R_{1}N_{2}]_{T} \\ -k_{on,V121,R1}^{T}[V_{121}]_{T}[R_{1}N_{2}]_{T}+k_{off,V121R1}^{T}[V_{121}R_{1}N_{2}]_{T} \end{matrix}$ (S.63)

$\begin{matrix} \frac{d[V_{121}R_{1}N_{2}]_{T}}{dt}=-k_{intV121R1N2}^{T}[V_{121}R_{1}N_{2}]_{T} \\ +k_{c,V121R1,N2}^{T}[V_{121}R_{1}]_{T,i}[N_{2}]_{T}-k_{dissoc,V121N2}^{T}[V_{121}R_{1}N_{2}]_{T} \\ +k_{on,V121R1N2}^{T}[V_{121}]_{T}[R_{1}N_{2}]_{T}-k_{off,V121R1N2}^{T}[V_{121}R_{1}N_{2}]_{T} \end{matrix}$ (S.64)

$\begin{matrix} \frac{d[A]_{T}}{dt}=-k_{on,V165,A}^{T}[V_{165}]_{T}[A]_{T}+k_{off,V165A}^{N}[V_{165}A]_{T} \\ -k_{on,V121,A}^{T}[V_{121}]_{T}[A]_{T}+k_{off,V121A}^{N}[V_{121}A]_{T} \\ +k_{pA}^{BT}\frac{S_{TB}}{U_{T}}\frac{U_{B}}{U_{P}}[A]_{B}-\left(\frac{k_{L}+k_{pA}^{TB}S_{TB}}{U_{T}}\right)\frac{[A]_{T}}{K_{AV,T}} \end{matrix}$ (S.65)

$\begin{matrix} \frac{d[V_{165}A]_{T}}{dt}=k_{on,V165,A}^{T}[V_{165}]_{T}[A]_{T}-k_{off,V165A}^{N}[V_{165}A]_{T} \\ +k_{pA}^{BT}\frac{S_{TB}}{U_{T}}\frac{U_{B}}{U_{p}}[V_{165}A]_{B}-\left(\frac{k_{L}+k_{pA}^{TB}S_{TB}}{U_{T}}\right)\frac{[V_{165}A]_{T}}{K_{AV,T}} \end{matrix}$ (S.66)

$\begin{matrix} \frac{d[V_{121}A]_{T}}{dt}=k_{on,V121,A}^{T}[V_{121}]_{T}[A]_{T}-k_{off,V121A}^{T}[V_{121}A]_{T} \\ +k_{pA}^{BT}\frac{S_{TB}}{U_{T}}\frac{U_{B}}{U_{p}}[V_{121}A]_{B}-\left(\frac{k_{L}+k_{pA}^{TB}S_{TB}}{U_{T}}\right)\frac{[V_{121}A]_{T}}{K_{AV,T}} \end{matrix}$ (S.67)

**GLOSSARY**

*Concentrations*

*[V_121_], [V_165_]* Concentration of unbound VEGF_121_ and VEGF_165_

*[M_ECM_], [M_EBM_], [M_PBM_]* Concentration of VEGF binding sites in the ECM, EBM, and PBM

*[V_165_M_ECM_], [V_165_M_EBM_], [V_165_M_PBM_]* Concentration of VEGF_165_ bound to the ECM, EBM, and PBM

*[R­_1_], [R_2_]* Concentration of un-occupiedVEGFR-1 and VEGFR-2 receptor tyrosine kinases

*[N_1_]* Concentration of un-occupied NRP1 co-receptor

*[N_2_]* Concentration of un-occupied NRP2 co-receptor

*[R_1_N_1_]* Concentration of the VEGFR1-NRP1 complex

*[R_1_N_2_]* Concentration of the VEGFR1-NRP2 complex

*[V_i_R_j_]* Concentration of VEGF isoform *i* bound to VEGFR *j*

*[V_i_N_1_]* Concentration of VEGF isoform *i* bound to NRP1

*[V_i_N_2_]* Concentration of VEGF isoform i bound to NRP2

*[R_2_V_165_N_1_]* Concentration of the VEGFR2-VEGF_165_-NRP1 ternary complex

*[R_2_V_165_N_2_]* Concentration of the VEGFR2-VEGF_165_-NRP2 ternary complex

*[V_121_R_1_N_1_]* Concentration of the VEGF_121_-VEGFR1-NRP1 ternary complex

*[V_121_R_1_N_2_]* Concentration of the VEGF_121_-VEGFR1-NRP2 ternary complex

*[A]* Concentration of anti-VEGF agent

*[V_i_A]* Concentration of VEGF isoform *i* bound to anti-VEGF agent

*Geometric parameters*

*U_i_* Volume of compartment *i* (*N*=normal tissue, *B*=blood, *P*=plasma, *T*=tumor)

*S_iB_* Total surface area of endothelial cells at the interface of compartment *i* and blood (*N*=normal tissue, *T*=tumor)

*K_AV,i_* Available volume fraction in the tissue, i.e., ratio of available fluid volume to total tissue volume *U_i_*

*Kinetic parameters*

*q_V121_, q_V165_* Secretion rate of VEGF_121_ and VEGF_165_

*q_A_* Injection rate of exogenous anti-VEGF agent; $q_{A}$ = 7.78 mg/min during the infusion time (90 minutes) and $q_{A}$ = 0 at all other times.

*s_R_* Insertion rate of receptors into the cell membrane of endothelial cells or myocytes

*k_on_* Kinetic binding rate

*k_off_* Kinetic unbinding rate

*k_c_* Kinetic coupling rate for receptors

*k_int_* Internalization rate of receptors

$k_{pV}^{ij}$ Microvascular permeability of VEGF from compartment *i* to compartment *j* (*N*=normal tissue, *B*=blood, *T*=tumor)

$k_{pA}^{ij}$ Microvascular permeability of anti-VEGF agent and VEGF/anti-VEGF complex from compartment *i* to compartment *j* (*N*=normal tissue, *B*=blood, *T*=tumor)

*k_L_* Lymphatic drainage rate

*c_V121_, c_V165_* Rate of plasma clearance of VEGF_121_ and VEGF_165_

*c_A_, c_V121A_, c_V165A_* Rate of plasma clearance of anti-VEGF and VEGF/anti-VEGF complex
