# Supplementary figures and images for: Pharmacokinetics and pharmacodynamics of VEGF-neutralizing antibodies
Source: BMC Syst Biol. 2011 Nov 21;5:193. doi: 10.1186/1752-0509-5-193 (PMC3229549; doi:10.1186/1752-0509-5-193)

**A**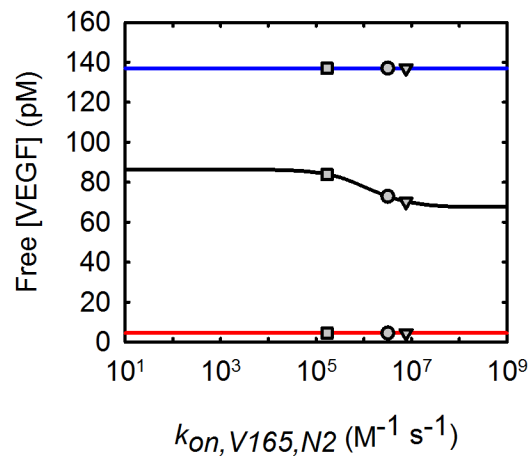**C**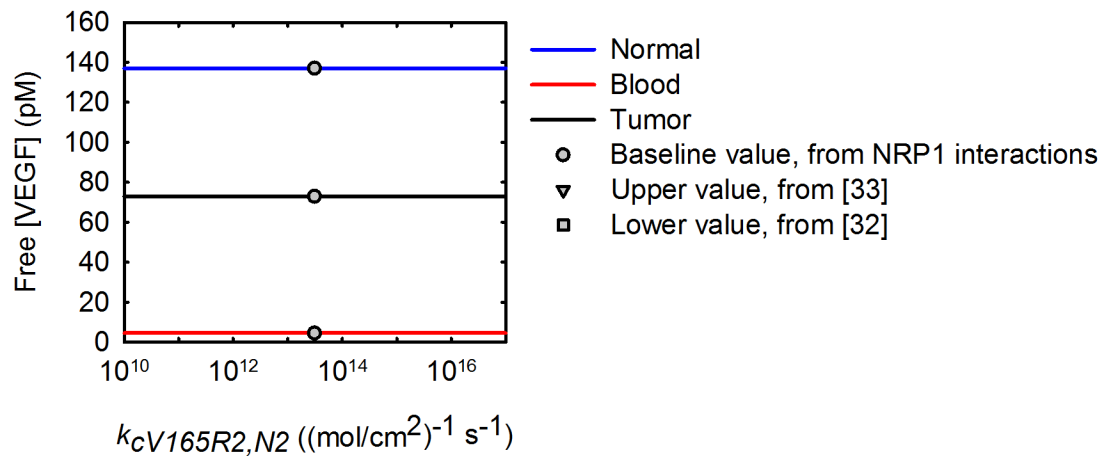**B**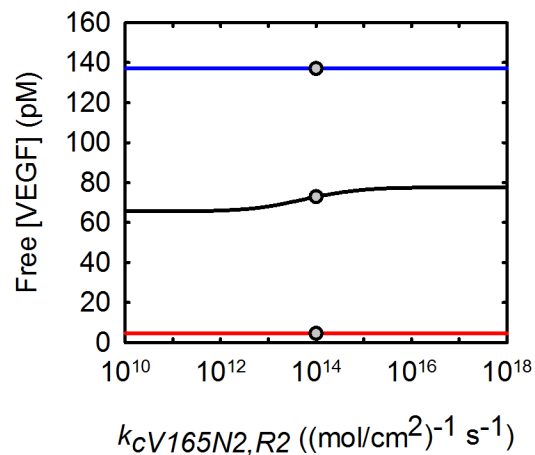**D**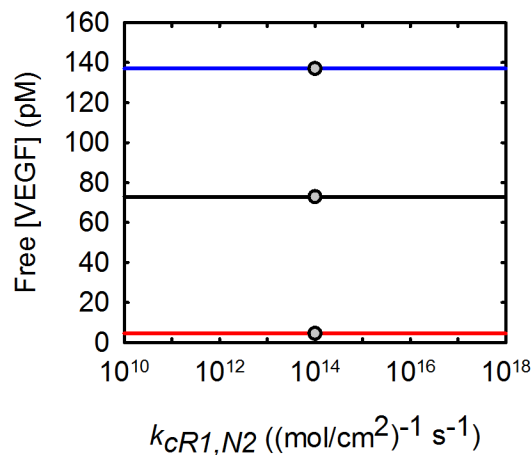

Supplement: Additional file 2 — We investigated how the kinetic parameters governing NRP2 binding to VEGF165 and coupling to VEGFR1 and VEGFR2 influenced the steady-state concentration of free VEGF in the body. A, Effect of VEGF165 binding to NRP2; squares and triangles indicate lower and upper values of kon, respectively, taken from literature. B, Effect of NRP2 coupling to VEGF165/VEGFR2. C, Effect of VEGF165/NRP2 coupling to VEGFR2. D, Effect of NRP2 coupling to VEGFR1. In all panels, gray circles indicate baseline values taken from NRP1 interactions. [file 1752-0509-5-193-S2.PDF]

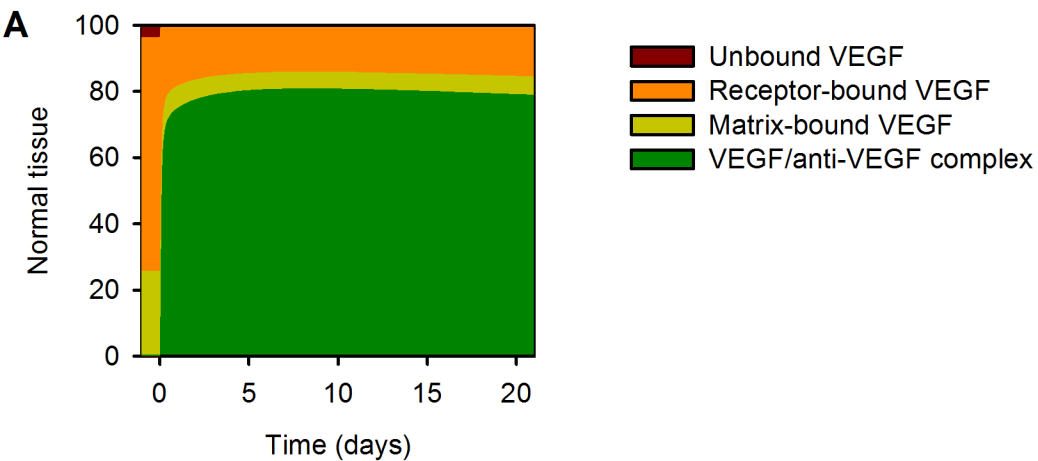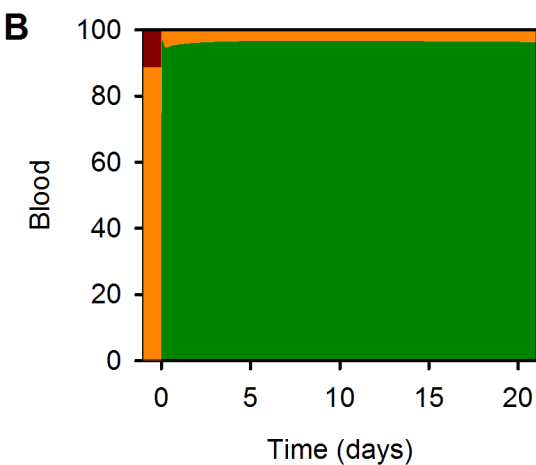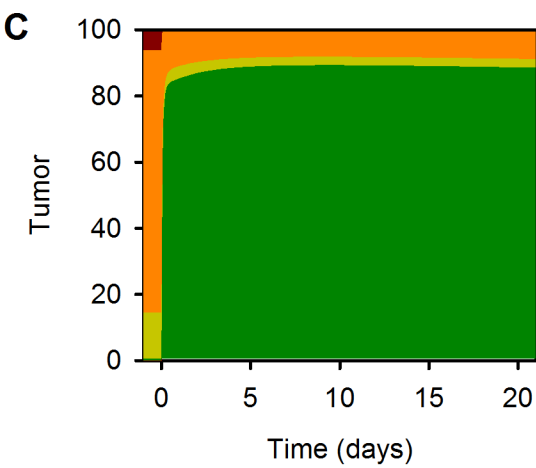

Supplement: Additional file 4 — VEGF distribution following a single intravenous injection of 10 mg/kg of anti-VEGF given at time 0. A, normal tissue, B, blood, and C, tumor. [file 1752-0509-5-193-S4.PDF]

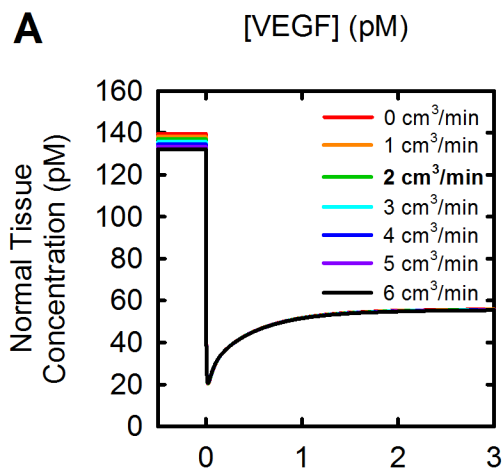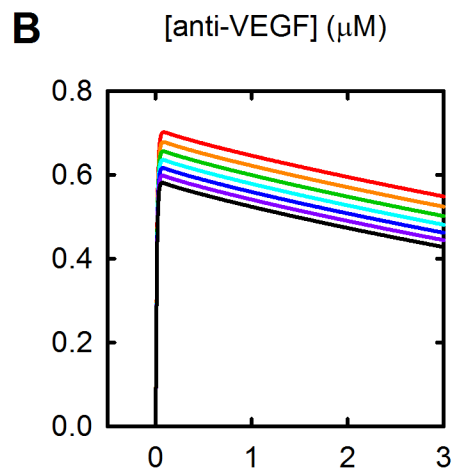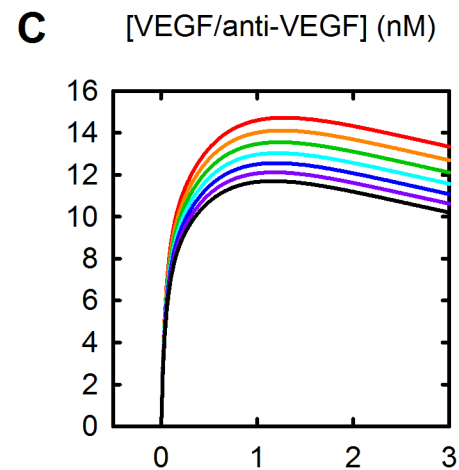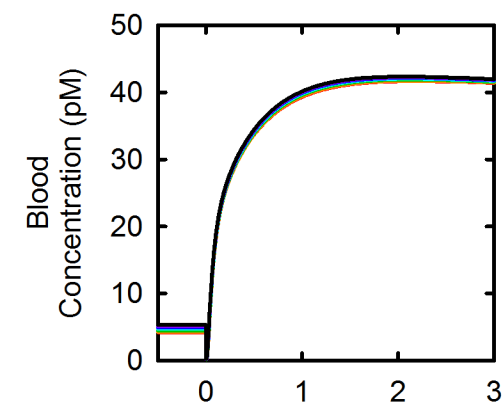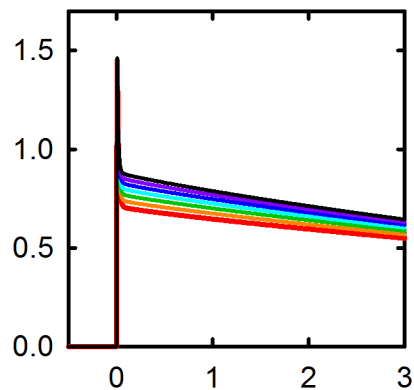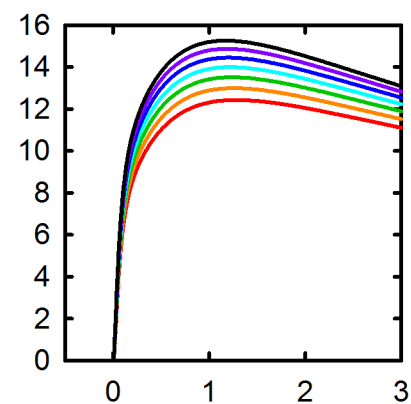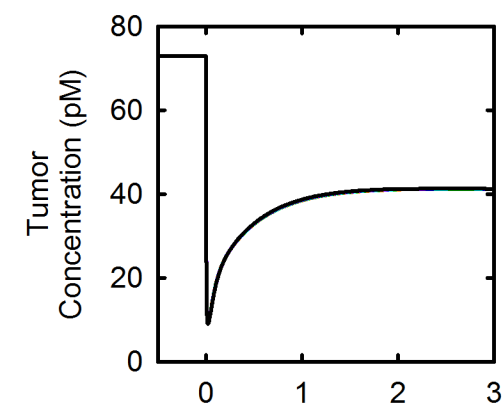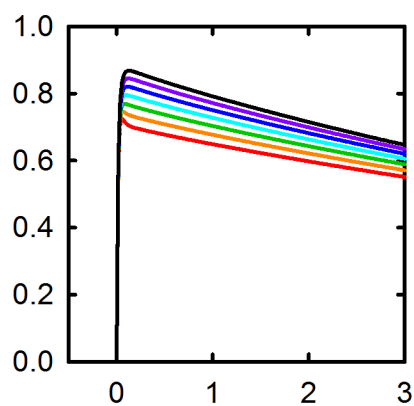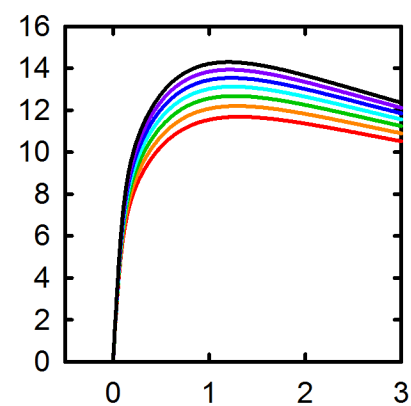

Time (weeks)

Time (weeks)

Time (weeks)

Supplement: Additional file 5 — The concentration profiles for A, free VEGF; B, free anti-VEGF; and C, the VEGF/anti-VEGF complex are predicted as the lymphatic flow rate from the normal tissue to the blood was varied. From top to bottom: normal tissue, blood, and tumor. The lymph flow rate influences the concentration of the anti-VEGF and VEGF/anti-VEGF complex. Legend in A applies to all panels; bold in the legend indicates the parameter value used in the current model. [file 1752-0509-5-193-S5.PDF]

**A** $k_{p,AV}^{NB}$ 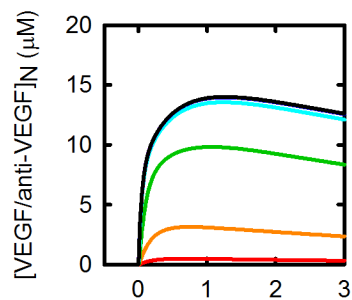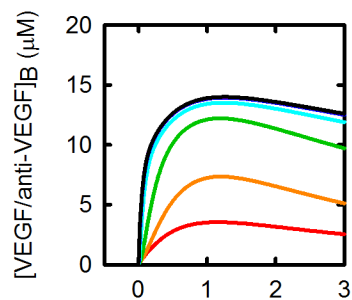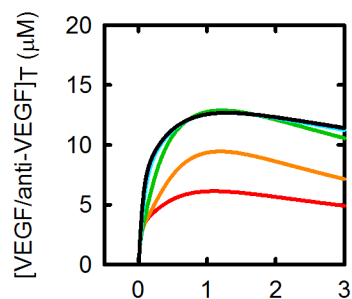

Time (weeks)

 $k_{p,AV}^{NB}$ 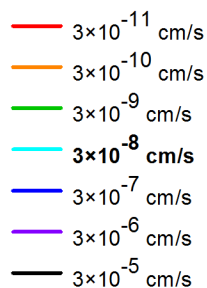**B** $k_{p,AV}^{TB}$ 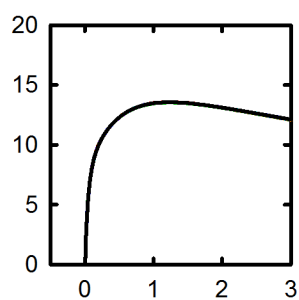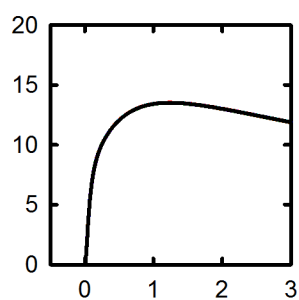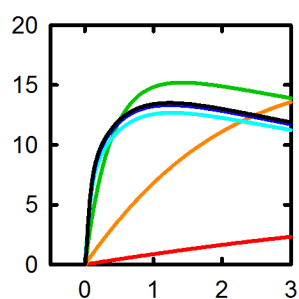

Time (weeks)

 $k_{p,AV}^{TB}$ 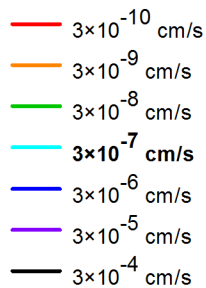**C** $k_c$ 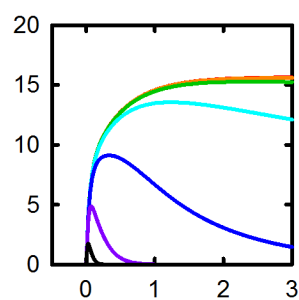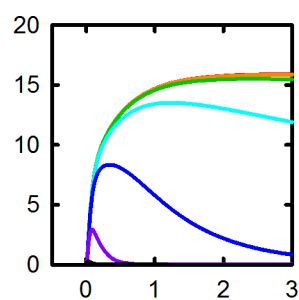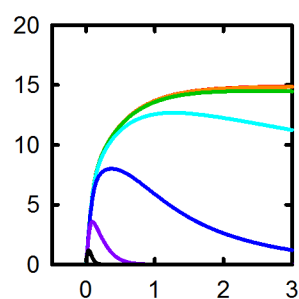

Time (weeks)

 $k_c$ 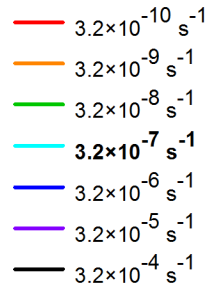**D** $K_d$ 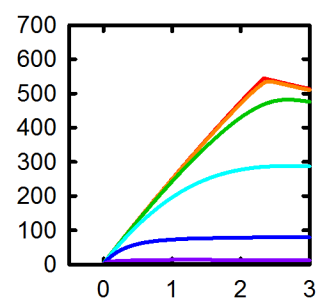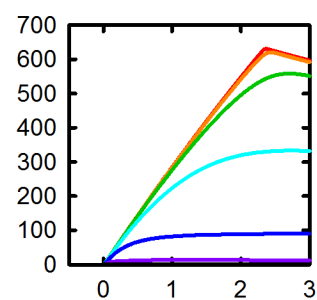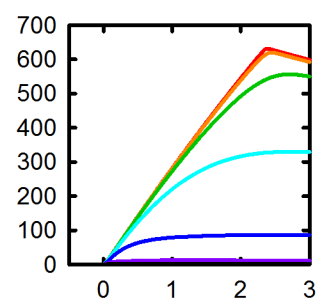

Time (weeks)

 $K_d$ 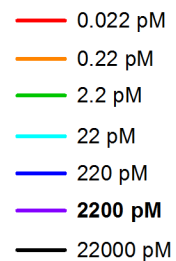

Supplement: Additional file 6 — The concentration of the VEGF/anti-VEGF complex in the body following anti-VEGF treatment is predicted as properties of the anti-VEGF are varied. A, Effect of microvascular permeability to anti-VEGF between the normal tissue and blood. B, Effect of microvascular permeability to anti-VEGF between the tumor and blood. C, Effect of clearance rate of anti-VEGF. D, Effect of anti-VEGF binding affinity to VEGF. From top to bottom: normal tissue, blood, and tumor. Bold in the legend indicates parameter value used in the current model. [file 1752-0509-5-193-S6.PDF]

**A** $k_{p,AV}^{NB}$ 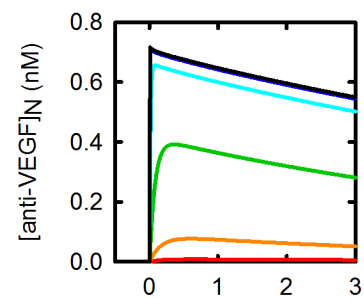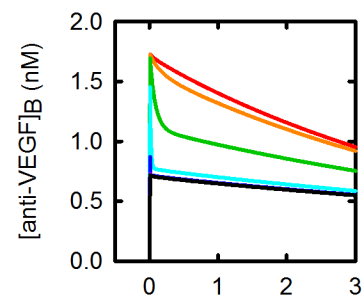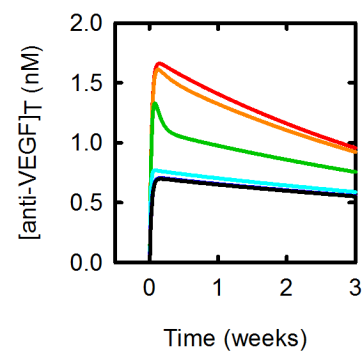**B** $k_{p,AV}^{TB}$ 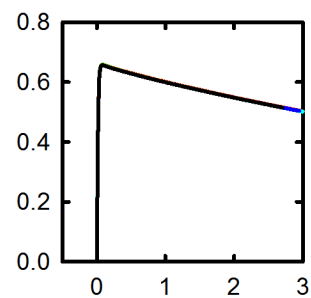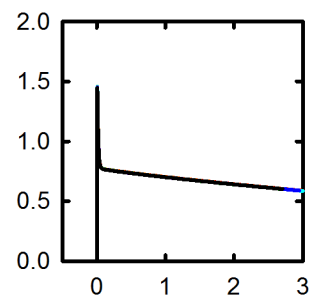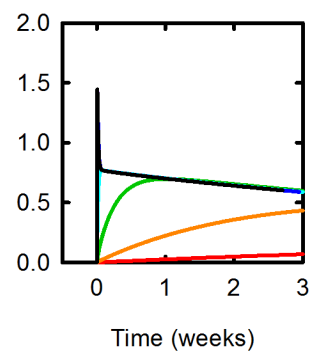**C** $k_c$ 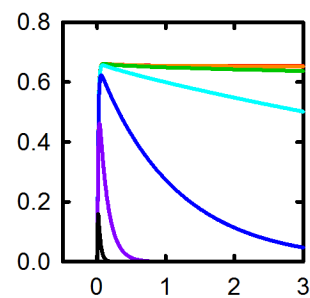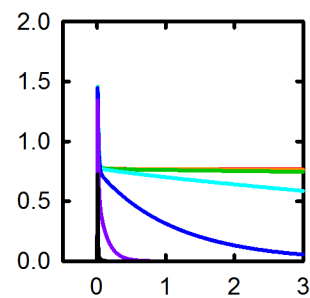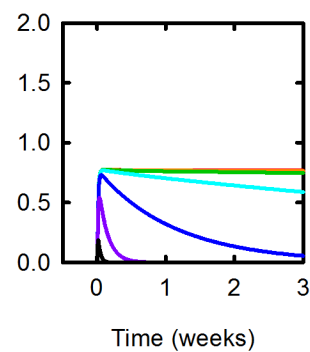**D** $K_d$ 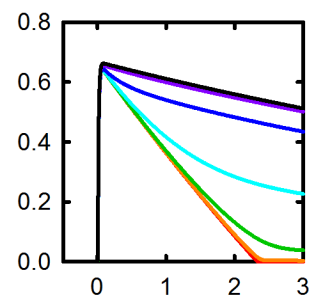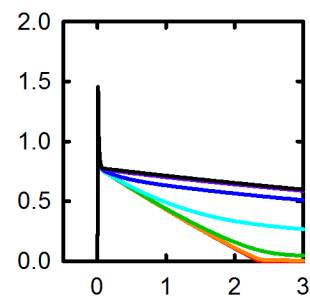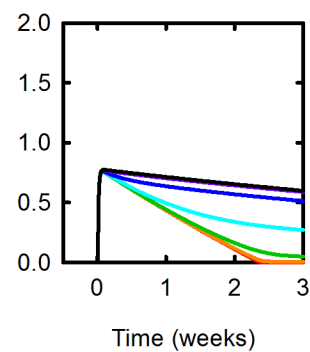 $k_{p,AV}^{NB}$ 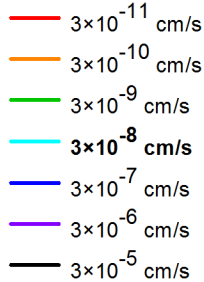 $k_{p,AV}^{TB}$ 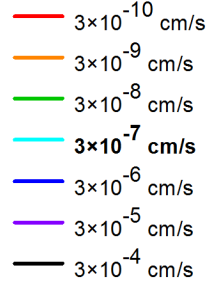 $k_c$ 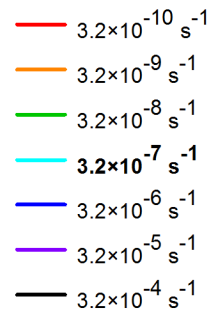 $K_d$ 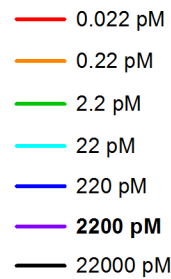

Supplement: Additional file 7 — The concentration of free anti-VEGF in the body following anti-VEGF treatment is predicted as properties of the anti-VEGF are varied. A, Effect of microvascular permeability to anti-VEGF between the normal tissue and blood. B, Effect of microvascular permeability to anti-VEGF between the tumor and blood. C, Effect of clearance rate of anti-VEGF. D, Effect of anti-VEGF binding affinity to VEGF. From top to bottom: normal tissue, blood, and tumor. Bold in the legend indicates parameter value used in the current model. [file 1752-0509-5-193-S7.PDF]
