# Supplementary material for: Pharmacokinetics and pharmacodynamics of VEGF-neutralizing antibodies
Source: BMC Syst Biol. 2011 Nov 21;5:193. doi: 10.1186/1752-0509-5-193 (PMC3229549; doi:10.1186/1752-0509-5-193)
Supplement: Additional file 3 — Comparison of the concentration of free VEGF in the tumor interstitial space calculated from experimental data and based on model predictions. [file 1752-0509-5-193-S3.DOC]

**Comparison of experimental and predicted free VEGF concentration in the tumor interstitial space**

*Quantitative tumor VEGF measurements*

We have compared the free VEGF level in the tumor predicted by our model to published quantitative data from a meta-analysis that provides a comprehensive literature review of VEGF concentration in human tumor tissue for various cancer types [1].

*Calculation of tumor VEGF*

The meta-analysis performed by Kut and coworkers determines the weighted average of VEGF in the tumor for all cancer studies to be 334 pg/mg protein. The concentration of VEGF in the tumor is estimated using a protein mass ratio of 154 mg protein/g fat-free tissue and tissue density of 1.06 g tissue/mL [1]. Using these conversion factors, tumor VEGF is calculated to be 54,611 pg/mL

*Calculation of tumor free VEGF*

The measurement described above is for total VEGF; however our model predicts the level of free VEGF, which is portion of the total VEGF in the tumor. Therefore in order to directly compare to our model predictions, we must assume that a percentage of the total VEGF determined in the experimental measurements is unbound. Figure S2-1 illustrates the concentration of unbound VEGF calculated from the experimental data compiled by Kut *et al.*, assuming different percentages of total VEGF, along with the predicted concentration of free VEGF from the current model.

Figure S2‑1: Comparison of predicted and experimental free VEGF concentration in the tumor. Free VEGF in the tumor is estimated from experimental data, assuming unbound VEGF comprises 1-6% of total VEGF. Blue circles indicate data from Kut and coworkers; gray dashed line indicates concentration of free VEGF predicted by the current model.

The predicted level of free VEGF is within the range provided by the experimental data. If we assume that 6% of the total VEGF is unbound, as our model predicts, the predicted concentration of free VEGF is 3.4-fold less than the concentration determined from the experimental data obtained from the meta-analysis.

**References**

1. Kut C, Mac Gabhann F, Popel AS**: Where is VEGF in the body? A meta-analysis of VEGF distribution in cance**r*. Br J Cance*r 2007**, 9**7:978-985.
